# Sensitivity of cells to ATR and CHK1 inhibitors requires hyperactivation of CDK2 rather than endogenous replication stress or ATM dysfunction

**DOI:** 10.1038/s41598-021-86490-x

**Published:** 2021-03-29

**Authors:** Jennifer P. Ditano, Katelyn L. Donahue, Laura J. Tafe, Charlotte F. McCleery, Alan Eastman

**Affiliations:** 1 Department of Molecular and Systems Biology, Geisel School of Medicine at Dartmouth, Lebanon, NH 03756 USA; 2 Norris Cotton Cancer Center, Geisel School of Medicine at Dartmouth, Rubin Building Level 6, One Medical Center Drive, Lebanon, NH 03756 USA; 3 Department of Pathology, Dartmouth Hitchcock Medical Center, Lebanon, NH 03756 USA

**Keywords:** Cancer, Cell biology

## Abstract

DNA damage activates cell cycle checkpoint proteins ATR and CHK1 to arrest cell cycle progression, providing time for repair and recovery. Consequently, inhibitors of ATR (ATRi) and CHK1 (CHK1i) enhance damage-induced cell death. Intriguingly, both CHK1i and ATRi alone elicit cytotoxicity in some cell lines. Sensitivity has been attributed to endogenous replications stress, but many more cell lines are sensitive to ATRi than CHK1i. Endogenous activation of the DNA damage response also did not correlate with drug sensitivity. Sensitivity correlated with the appearance of γH2AX, a marker of DNA damage, but without phosphorylation of mitotic markers, contradicting suggestions that the damage is due to premature mitosis. Sensitivity to ATRi has been associated with ATM mutations, but dysfunction in ATM signaling did not correlate with sensitivity. CHK1i and ATRi circumvent replication stress by reactivating stalled replicons, a process requiring a low threshold activity of CDK2. In contrast, γH2AX induced by single agent ATRi and CHK1i requires a high threshold activity CDK2. Hence, phosphorylation of different CDK2 substrates is required for cytotoxicity induced by replication stress plus ATRi/CHK1i as compared to their single agent activity. In summary, sensitivity to ATRi and CHK1i as single agents is elicited by premature hyper-activation of CDK2.

## Introduction

Genomic instability is a hallmark of cancer, and results from a high rate of spontaneous DNA damage or defects in DNA repair. The underlying mechanism of genomic instability (e.g., DNA repair deficiency or replicative stress) can provide an Achilles heel through which novel and selective therapeutic strategies can be designed. Inhibitors of poly(ADP-ribose) polymerase (PARP) have pioneered this chemical-genetic synthetic lethal approach because they selectively inhibit tumors deficient in BRCA1, BRCA2 or other proteins involved in homologous recombination repair^1–3^.

DNA damage elicits two parallel and interacting pathways to elicit cell cycle arrest and repair. Stalled replication forks generate single-strand regions of DNA (ssDNA) that activate ATR and subsequently CHK1. DNA double-strand breaks (DSB) recruit the MRN complex (Mre11, Rad50, Nbs1), which in turn recruits and activates ATM and subsequently CHK2. Mre11 possesses a nuclease activity that contributes to the generation of single-strand regions at DSB which in turn recruit and activate ATR/CHK1. The consequence of activating CHK1/2 is that they phosphorylate and inhibit CDC25 phosphatases thereby preventing activation of CDK1/2 resulting in cell cycle arrest at either S or G_2_ phase. CHK1 inhibitors (CHK1i) were initially developed as a means to circumvent this arrest, force premature cell cycle progression and enhance the cytotoxicity of DNA damaging therapies^4^.

Intriguingly, some cell lines are very sensitive to CHK1i as monotherapy. We recently published a study of the response of 65 human cell lines to the CHK1 inhibitor MK-8776; about 15% of these cell lines are hypersensitive to MK-8776, resulting in ssDNA and DSB that occur after only a short incubation time (6 h)^5^. MK-8776 still inhibited CHK1 in the resistant cells, but they could tolerate this inhibition for at least 7 days while continuing to proliferate. If 15% of human tumors are also hypersensitive to CHK1i, this likely represents a chemical-genetic synthetic lethal interaction that can be therapeutically targeted. Identification of the defects that sensitize tumor cells to CHK1i would facilitate patient stratification and provide precision medicine for patients with such tumors.

We recently demonstrated that sensitivity to CHK1i resulted from aberrant hyper-activation of CDK2 in early S phase, followed by the phosphorylation of H2AX (γH2AX)^4,5^. Furthermore, this sensitivity is dependent on cyclin A rather than cyclin E^5^. In an attempt to further understand the mechanism of sensitivity to CHK1i, we have now performed a comparative analysis of the sensitivity to inhibition of the upstream regulator of CHK1, namely ATR. It has been proposed that sensitivity to both CHK1i and ATR inhibitors (ATRi) depends on endogenous replicative stress^6–10^, yet this is inconsistent with the very different pattern of sensitivity we observe for these two drugs. Replication stress has been defined as slowing or stalling of replication fork progression that usually results in stretches of ssDNA, and is often observed as constitutive activation of the DNA damage response^7,11^. Here, we survey our panel of cell lines for constitutive activation of the DNA damage response and find this also does not correlate with sensitivity to either CHK1i or ATRi. Loss of function of ATM has often been associated with sensitivity to ATRi^12–15^, and we find this to be true in an isogenic background. However, many ATM mutations do not appear to generate ATM dysfunction, while some cell lines are sensitive to ATRi with no known defect in the ATM pathway. Of particular note, cells with low levels of ATM may still be fully functional for ATM signaling, and therefore assessment of ATM levels may mislead attempts to stratify patients for those who may respond to treatment with an ATRi. Finally, we establish that sensitivity to ATRi, like sensitivity to CHK1i, depends on hyperactivation of CDK2.

## Results

### Sensitivity to ATRi does not correlate with sensitivity to CHK1i

**Figure 1:**
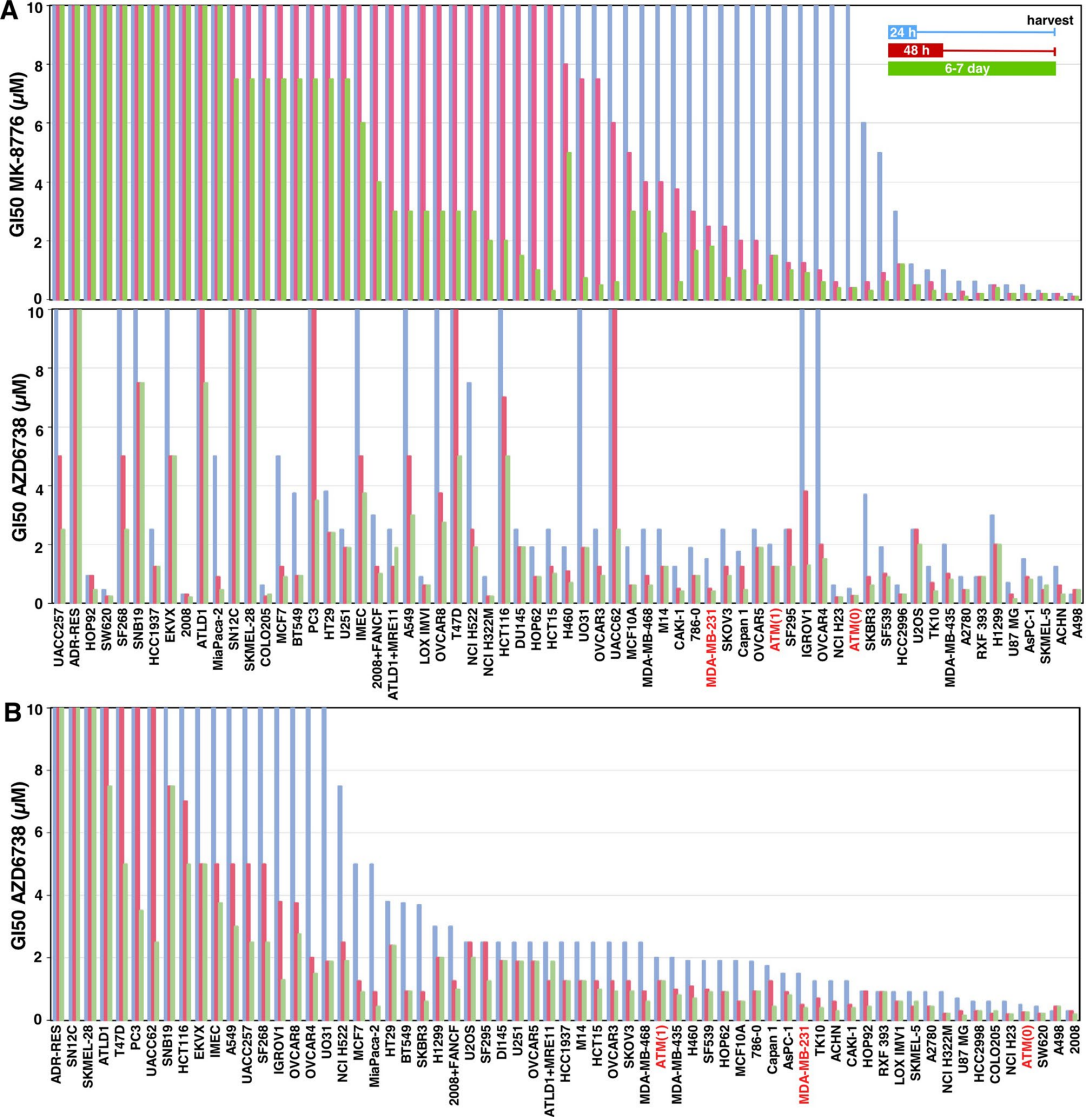
Sensitivity of cell lines to MK-8776 and AZD6738. (**A**) Each cell line was incubated with MK-8776 (top) or AZD6738 (bottom) for either 24 h or 48 h, then drug was removed, and cells were incubated in fresh media for an additional 5–6 days. Alternately, cells were incubated in drug continuously for 6–7 days. Cells were lysed in the well, stained with Hoechst 33258, and the concentration that inhibited growth by 50% was recorded. Cell lines in both panels are sorted by sensitivity to MK-8776. The panel for MK-8776 is updated from our prior paper^5^. (**B**) Results for AZD6738 from A are resorted in order of sensitivity to AZD6738. The cell lines highlighted in red are MDA-MB-231 and its ATM-dysfunctional derivatives discussed later.

Having previously established a large difference in sensitivity to the CHK1i MK-8776 across multiple cell lines^5^, this study began with a parallel assessment of the sensitivity of the cells to the ATRi AZD6738. Three different exposure times with drug were used: 24 h, 48 h or 7 days. After the shorter incubations, drug was removed and cells incubated in complete medium until almost confluent (an additional 5–6 days). The different incubation times provide information on the exposure time required for growth inhibition, and the potential for recovery following brief drug treatment. The short drug exposure times are also more relevant to the administration schedule used in patients. The concentration that inhibited growth by 50% (GI50) was then calculated and compared to the sensitivity to CHK1i (Fig. 1A). The data is also presented in the order of sensitivity to AZD6738 to emphasize three levels of sensitivity: about a quarter of the cell lines have GI50 values < 1 µM; about 40% have GI50 values around 2 µM; the remainder are relatively insensitive (Fig. 1B). The full set of values is presented in a spread sheet (Supplementary Table 1).

**Figure 2:**
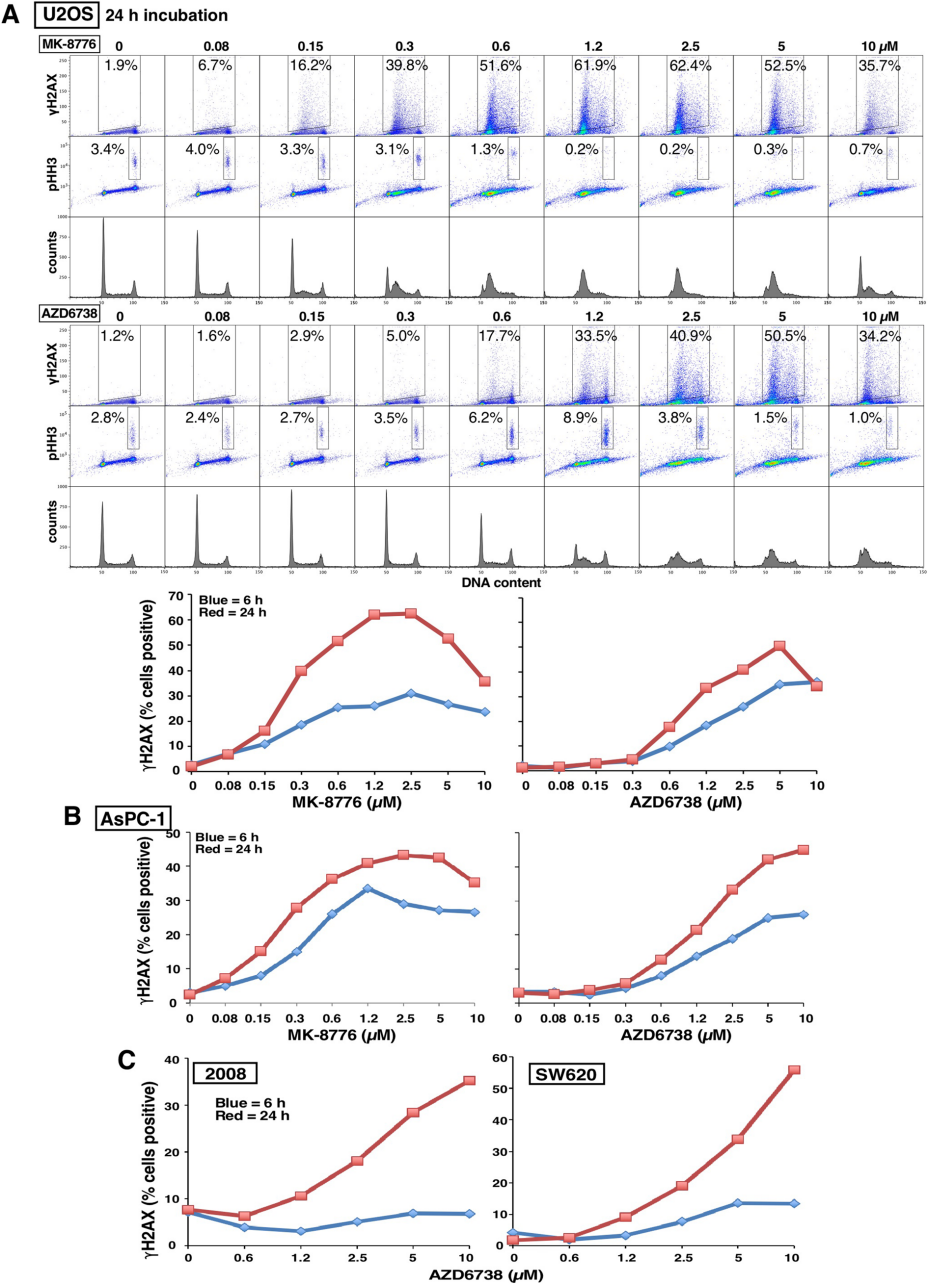
Induction of γH2AX by MK-8776 and AZD6738 in U2OS and AsPC-1 cells. (**A**) U2OS cells were incubated with the indicated concentrations of MK-8776 (upper panel) or AZD6738 (lower panel) for 6 h (not shown) or 24 h, then fixed and analyzed by flow cytometry for DNA content, γH2AX and pHH3. The percentage of cells positive for γH2AX or pHH3 is shown, and the results for γH2AX-positive cells are graphed. (**B**) ASPC-1 cells were incubated with MK-8776 and AZD6738 and analyzed by flow cytometry as in A. (**C**) 2008 and SW620 cells were incubated with AZD6738 for 6 or 24 h and analyzed as in A. The original data for panels B and C are presented in Supplementary Fig. 1.

It is apparent that many more cell lines are sensitive to ATRi than CHK1i (Fig. 1). Since the cell lines previously shown to be sensitive to CHK1i were also sensitive to ATRi, we investigated further similarities between the two. Sensitivity to CHK1i is associated with rapid appearance of γH2AX^5^, but it was previously reported that sensitive cells exhibited little γH2AX when incubated with ATRi^16^. However, that analysis was performed solely in U2OS cells and with only a single concentration of each drug. In our hands, U2OS cells are several-fold less sensitive to AZD6738 than to MK-8776, and they exhibit a similar level of γH2AX at equitoxic concentrations (Fig. 2A). A parallel experiment in another sensitive cell line, AsPC-1, also showed that a slightly higher concentration of AZD6738 than MK-8776 was required to induce γH2AX consistent with the difference in cytotoxicity (Fig. 2B; Supplementary Fig. 1). Furthermore, γH2AX occurred primarily in early S phase cells, rather than in G2/M as previously reported^17^, and there was no concurrent increase in the mitotic marker phospho-histone H3 (pHH3). This lack of correlation between γH2AX and pHH3 contradicts the prior suggestion that premature mitosis induced by either CHK1i or ATRi leads to γH2AX^17,18^.

Many of the cell lines were sensitive to ATRi but remained insensitive to CHK1i, for example, SW620 and 2008 (Fig. 1). We therefore determined whether these cells induced γH2AX when incubated with ATRi. SW620 and 2008 cells demonstrated limited γH2AX within 6 h, but when the incubation was extended to 24 h, considerably more γH2AX was detected (Fig. 2C; Supplementary Fig. 1). The 24-h incubations also demonstrate that cells continue to progress into S phase while exposed to AZD6738. While most of the γH2AX in 2008 cells occurred in S phase, in the SW620 cells, the γH2AX-positive cells were primarily in late S or G2. As observed with U2OS and AsPC-1 cells, there was still little increase in pHH3 suggesting that there is no premature mitosis.

### Resistance to ATRi is not due to drug metabolism or reduced drug uptake

**Figure 3:**
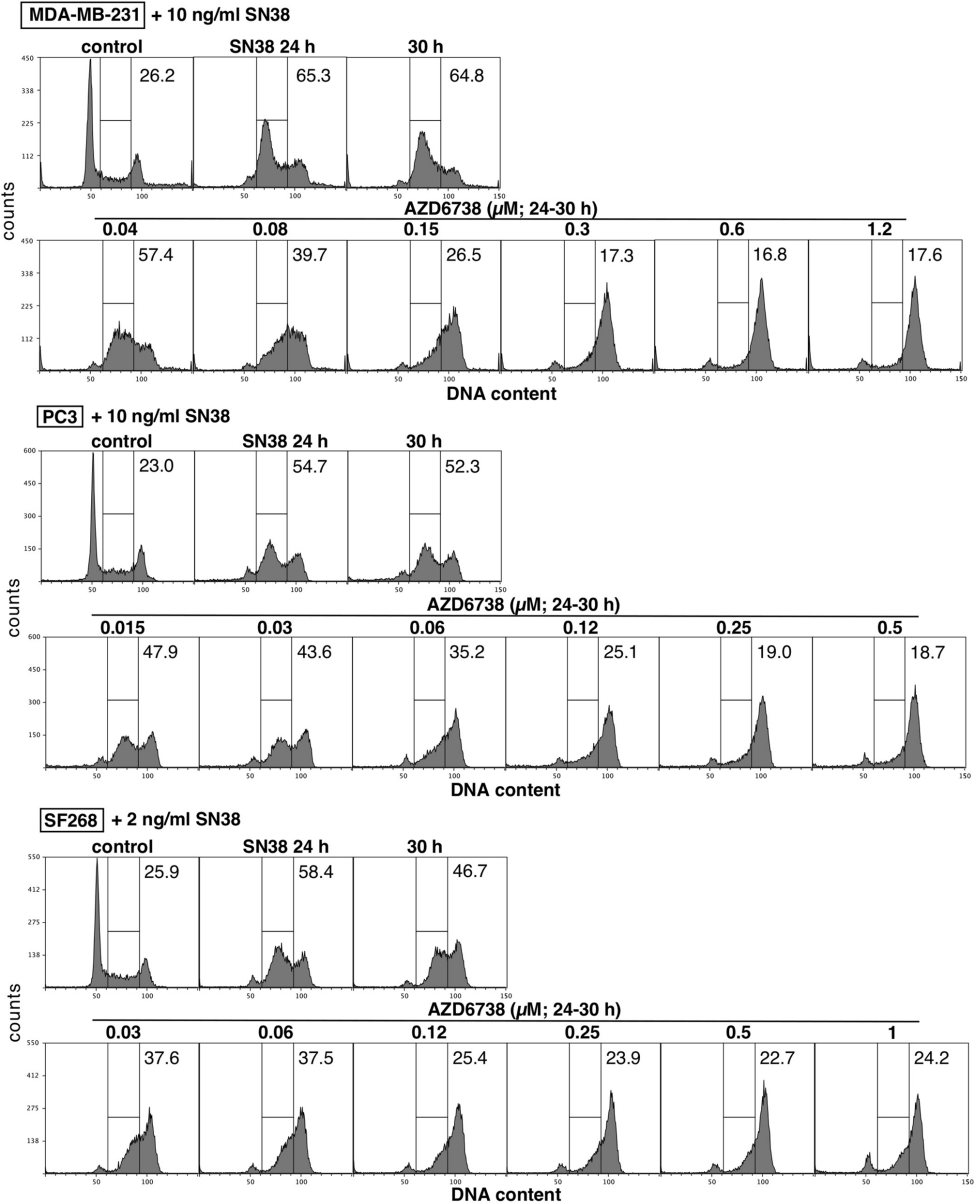
Abrogation of DNA damage-induced cell cycle arrest by AZD6738 in sensitive and resistant cell lines. MDA-MB-231 (sensitive), PC3 (resistant) and SF268 (resistant) cells were incubated with the indicated concentrations of SN38 for 24 h, then SN38 was removed. Cells were then incubated with the indicated concentrations of AZD6738 from 24 to 30 h, fixed and analyzed by flow cytometry for DNA content. Numbers represent the percentage of cells in S phase. The top row for each cell line shows untreated cells, cell treated with only SN38 for 24 h, and cells after an additional 6 h in media.

One mechanism to elicit resistance to ATRi would be to alter cellular pharmacology of the drug through uptake or metabolism. We therefore assessed the ability of ATRi to abrogate cell cycle arrest induced by the topoisomerase I inhibitor SN38. Using this approach, we have previously noted that cells resistant to CHKi still abrogate S phase arrest induced by SN38 when incubated with MK-8776^5^. We compared the response of MDA-MB-231 cells, which are relatively sensitive to ATRi, to two resistant lines, PC3 and SF268 (Fig. 3). A concentration of SN38 was selected that is known to arrest each cell line in S phase after a 24 h incubation^19^. SN38 was then removed, and ATRi was added for 6 h to assess their progression to G2. ATRi was equally effective at abrogating arrest in all three cell lines. Hence, the mechanism of resistance of PC3 and SF268 cells to ATRi as a single agent is not due to a failure of the drug to inhibit its target.

### Sensitivity to CHK1i or ATRi does not correlate with constitutive activation or defects in the DNA damage response pathway

**Figure 4:**
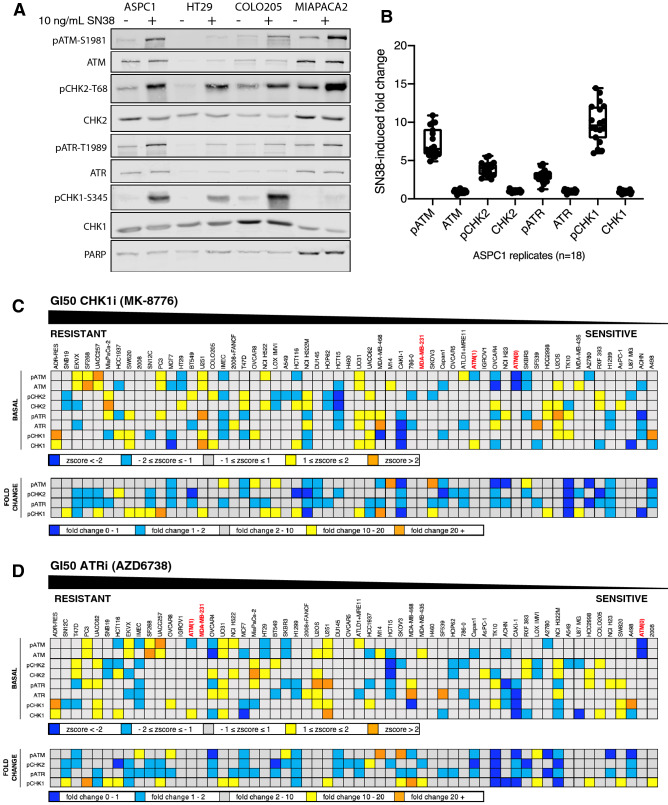
Relationship between sensitivity to MK-8776 or AZD6738 and endogenous DNA damage or DDR pathway function. (**A**) A representative western blot in which the internal standard cell line (AsPC-1) and additional cell lines (HT29, COLO205, MiaPaca2) were untreated or incubated with 10 ng/mL SN38 for 6 h. Cells were then rinsed with PBS, lysed and analyzed using the indicated primary antibodies and fluorescent secondary antibodies. The full set of western blots is shown in Supplementary Fig. 2. (**B**) Raw signal intensities of the indicated proteins were collected for each ASPC-1 internal standard replicate using Image Studio Lite, then used to calculate SN38-induced fold change. Replicate fold-change values for each protein were then analyzed in GraphPad Prism 8 to generate box-and-whisker plots. (**C**) Raw signal intensities of the indicated proteins were collected for all cell lines using Image Studio Lite, then normalized to the appropriate AsPC-1 internal standard. For basal protein expression (upper panel), AsPC-1-normalized values for each untreated sample were log-transformed and then used to calculate z-scores representing expression of each protein across all cell lines. For fold change (lower panel), AsPC-1-normalized values for untreated and SN38-treated samples for each cell line were used to calculate the fold change of each protein. Cell lines were sorted by MK-8776 GI50 (Fig. 1A). (**D**) The data in panel C is resorted by GI50 for AZD6738 (Fig. 1B).

While the differences in sensitivity to CHK1i and ATRi appear to rule out endogenous replication stress as the reason for sensitivity to both drugs, it could still explain the sensitivity to one or other drug. We therefore asked whether the cells expressed constitutive activation of the DNA damage response that might indicate replicative stress, and whether this correlated with response to either CHK1i or ATRi. Cell lysates were analyzed for basal levels of ATM, ATR, CHK1 and CHK2, and their phosphorylation indicating their respective activation status. In parallel, cells were incubated with SN38, a well-known activator of the DNA damage response, to determine whether the ATM-CHK2 and ATR-CHK1 pathways were functional. A sample western blot is shown in Fig. 4A, while the entire data set is shown in Supplementary Fig. 2. AsPC-1 cells were used as a reference on every western blot as they expressed positive signals for each protein; the reproducibility of the results in repeated analyses of AsPC-1 cells is presented in Fig. 4C. Confidence in the analysis is also reflected in the fact that our results for endogenous phosphorylation of CHK2 are in agreement with a prior report^20^. Protein expression in each cell line was normalized to the AsPC-1 sample on each western blot, and then expressed as z-scores reflecting the level of deviation from the mean of all cell lines for that antigen. The values in Fig. 4C,D are aligned relative to the sensitivity to CHK1i or ATRi as presented in Fig. 1.

A few cell lines expressed relatively high levels of basal phosphorylation of one or two proteins (orange or yellow, Fig. 4) suggesting endogenous DNA damage, although there was no clear trend toward sensitivity or resistance to either CHK1i or ATRi. Similarly, there was no clear trend for cells low in basal phosphorylation (dark and light blue) with resistance to either drug. There was no indication of cells high for all phosphoproteins, and rarely were both members of the ATM-CHK2 pathway or the ATR-CHK1 pathway concurrently high or low. Furthermore, there was no indication that a defect in the ATM-CHK2 pathway might be compensated for by an increase in the ATR-CHK1 pathway (or vice versa). CAKI1 cells are the closest example of this as they appear to have low phosphorylation of ATR and CHK1, although they still have average phosphorylation of ATM and CHK2. A more formal analysis of these comparisons versus growth inhibition is presented in Supplementary Fig. 3. Additional discussion regarding the levels of ATM relative to ATM mutations is presented below.

The concurrent analysis of SN38-induced phosphorylation suggested that some cell lines are defective for eliciting a DNA damage response (dark blue). The most obvious in this regard is TK10, while CAKI1 and H322M also exhibited limited response. One potential cause for this apparent defect is a lack of induction of DNA damage by SN38 in these cell lines, albeit we have previously reported that all these cell lines arrest in G2 at or below the concentration of SN38 used here, and most arrest in S at this concentration^19^. TK10 were one of 4 cell lines (the others being HCT116, HCC2998 and IGROV1) that only appeared to arrest in G2 suggesting a checkpoint defect, but CAKI1 and H322M were fully competent for S phase arrest^19^. These data provide interesting cell lines for study of the impact of specific defects in DNA damage response, but there is no correlation with response to CHK1i or ATRi.

### ATM deletion sensitizes to ATRi

Dysfunctional ATM has frequently been reported to explain sensitivity to ATRi, and cells with mutant ATM are thought to have low levels of ATM^12–14^. In Fig. 4, OVCAR4 was the only cell line that appeared to completely lack ATM, although 7 other lines had levels below one standard deviation of the mean (See Supplementary Table 1 for individual values). We used CRISPR-Cas9 to generate a derivative of MDA-MB-231 with a complete knockout of ATM. We initially targeted ATM exon 3 and obtained many clones with reduced ATM protein, but no complete loss of ATM. We selected the lowest expressing clone, termed 231 ATM(1), for further study. Western blot analysis revealed that these cells produce roughly 30% of the level in the parental cell line (Supplementary Fig. 4). Sequencing of exon 3 uncovered 5 unique ATM alleles, 4 of which contained insertions or deletions resulting in a frameshift mutation, and one that contained an in-frame deletion (MDA-MB-231 cells are aneuploid with a modal number of 64 chromosomes). The low expression of ATM protein observed, together with data presented below, suggests that the allele harboring the in-frame deletion is still functional.

To generate a complete ATM knockout, we engineered a second CRISPR guide targeting exon 6 and transfected it into the 231 ATM(1) cells. This resulted in a clone termed 231 ATM(0) that expressed no detectable ATM protein. Sequencing of exon 6 revealed 4 unique alleles, three of which contained insertions or deletions resulting in frameshift mutations, and one which contained an in-frame insertion (Supplementary Fig. 4). With the known loss of 4/5 alleles in the 231 ATM(1) background, we surmise that the complete loss of ATM protein observed in 231 ATM(0) cells is due to the combination of mutations at both exon 3 and exon 6.

**Figure 5:**
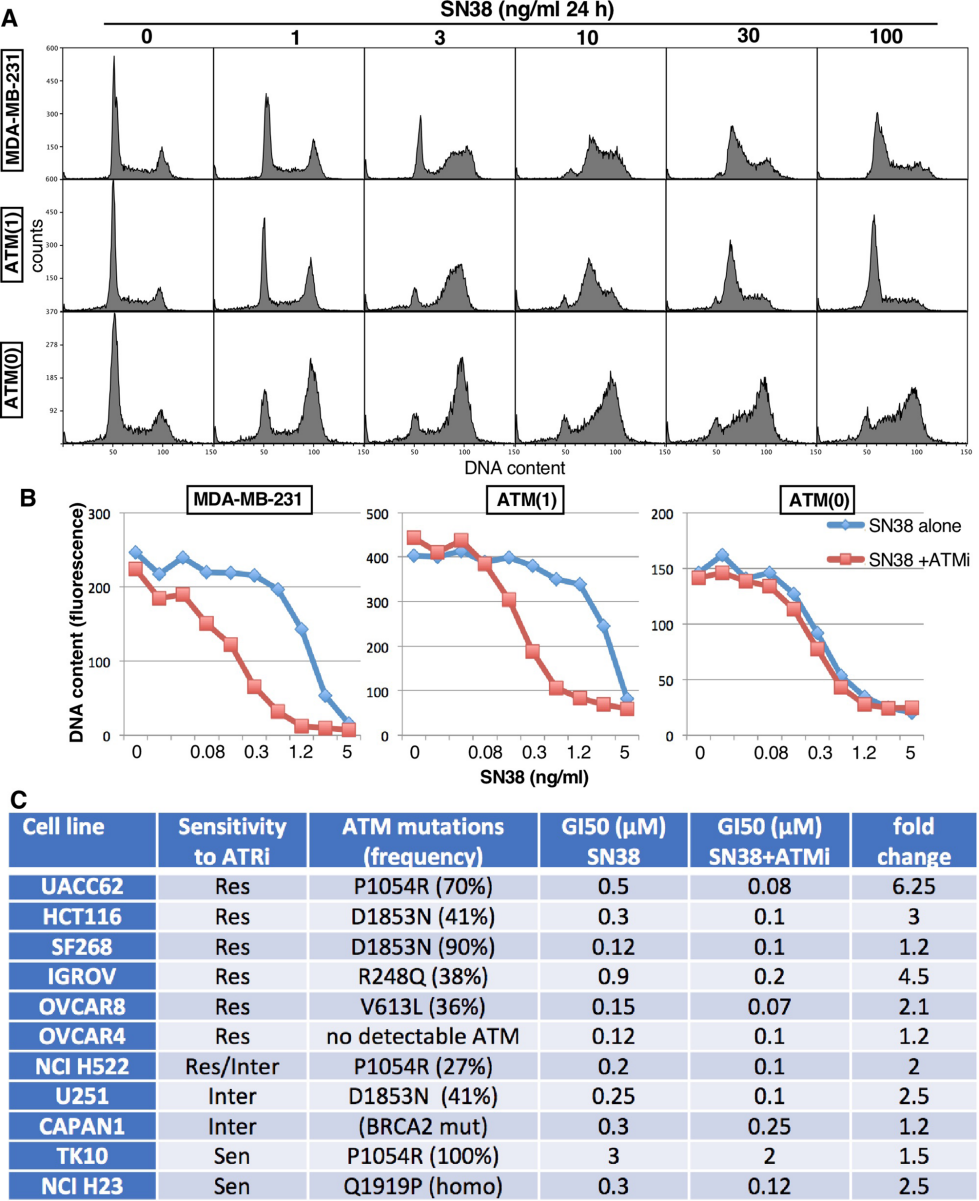
Impact of partial or complete ATM loss on S-phase arrest and sensitivity to combined SN38 and ATMi. (**A**) MDA-MB-231 wild-type, ATM(1), and ATM(0) cells were incubated with the indicated concentrations of SN38 for 24 h, then fixed and analyzed by flow cytometry for DNA content. (**B**) Cells were incubated with the 0–5 µM SN38 with or without 1 µM KU60019 (ATMi) for 24 h, drugs were removed and cells grown for an additional 6 days. Cells were fixed, DNA quantified and graphed versus SN38 concentration for each cell line. (**C**) Each cell line was incubated with SN38 with or without and ATMi as in B, and the GI50 concentrations assessed.

The ATM knockout cells were strikingly sensitive to ATRi (Fig. 1), and exhibited a checkpoint defect whereby they are far less efficient at arresting in S phase upon incubation with SN38, albeit they still arrested in G2 (Fig. 5A). This defect in S phase arrest is similar to that observed in TK10, HCT116, HCC2998 and IGROV1cells, yet the only cell line in the panel that expressed no ATM, OVCAR4, was still capable of exhibiting a normal S phase arrest in response to SN38^19^. The 231 ATM(1) cells with partial deletion of ATM were no more sensitive to ATRi (Fig. 1), and did not exhibit an S phase checkpoint defect when incubated with SN38 (Fig. 5A). This observation is important because it suggests low levels of ATM protein can still be fully functional.

### Sensitivity to ATRi does not correlate with dysfunction of ATM signaling

Many of the cell lines reported to have an ATM mutation (discover.nci.nih.gov/cellminer/) are relatively insensitive to ATRi, but major limitations of this analysis are that (a) some mutations are heterozygous and may not be dominant; (b) not all ATM mutations may be dysfunctional; and (c) there may be other defects in the ATM pathway that elicit sensitivity to ATRi. We therefore assayed for ATM function across these cell lines.

We previously reported that inhibition of ATM dramatically sensitizes cells to topoisomerase I inhibitors^21^, and this is consistent with the role of ATM in regulating removal of potentially lethal DNA-bound topoisomerase I complexes^22^. Cells deficient for ATM are expected to be sensitive to the topoisomerase I inhibitor SN38, and show no additional sensitivity when ATM is inhibited. This effect was observed when we compared MDA-MB-231 cells that were competent or deleted for ATM (Fig. 5B). The MDA-MB-231 ATM-wildtype cells, as well as the 231 ATM(1) cells, were relatively resistant to SN38, but were ~ eightfold more sensitive to SN38 when co-incubated with the ATMi KU60019. In contrast, the 231 ATM(0) cells were ~ fivefold more sensitive to SN38 and showed no further sensitization upon addition of ATMi.

We used this approach to assess the possible dysfunction of ATM in many of our cell lines and compared it to the cytotoxicity (Fig. 1) and protein levels (Fig. 4) described above. Detailed results are presented in Supplementary Table 1). Selected results are presented in Fig. 5C. The following are some of the observations from this analysis. Most of the mutations, their percent frequency, and SIFT/Polyphen scores are derived from the publicly available database (discover.nci.nih.gov/cellminer/).

- SF268 cells are relatively resistant to ATRi and have the highest level of ATM protein because of an amplified ATM gene (8.7-fold), however 90% is reportedly mutated at D1853N. These cells were very sensitive to SN38 with no further sensitization by ATMi. The D1853N mutation occurred in several other cell lines, but was always heterozygous and did not elicit ATM dysfunction. Hence, D1853N appears dysfunctional only if homozygous.
- HCT116 (resistant to ATRi) and U251 cells (intermediate sensitivity) are also reported to have a heterozygous D1853N mutation, and we have previously reported that both have very low MRE11 protein^19^, and it may be this latter modification that renders them sensitive to SN38 and only slightly more sensitive to the addition of ATMi.
- NCI H23 cells are homozygous at Q1919P (significant Polyphen score), sensitive to SN38 and only marginally impacted by ATMi. They are also very sensitive to ATRi.
- TK10 cells (sensitive to ATRi) are homozygous for P1054R mutation (significant SIFT and Poyphen scores), but also have the lowest level of ATM mRNA. While they are relatively resistant to SN38, they are insensitive to addition of ATMi. This suggests this mutation is dysfunctional.
- NCI H522 (fairly resistant to ATRi) have the same P1054R mutation but only at 27%, yet are very sensitive to SN38 and insensitive to ATMi, suggesting this mutation may be dominant. However, UACC62 cells (resistant to ATRi) express this mutation at 70% but are only slightly sensitive to SN38 and are still sensitive to ATMi. Hence, it appears that the lack of impact of ATMi in both H522 and TK10 cells more likely reflects an alternate defect in the ATM pathway.
- OVCAR8 (resistant to ATRi) have two heterozygous mutations in ATM (V613L and D1853N) and are very sensitive to SN38 and almost insensitive to ATMi. Whether one of these alleles is dominant or they are both dysfunctional but in different alleles is unknown.
- IGROV (resistant to ATRi) have a R248Q mutation with significant SIFT and Polyphen scores but the mutation is heterozygous and the cells are not very sensitive to SN38 and they are sensitized by ATMi.
- Several other cell lines were also insensitive to ATMi, but with no know mutation in ATM, suggesting alternate mutations in this damage response pathway may exist. This was true of CAPAN1 cells that are defective for BRCA2, sensitive to SN38 and unaffected by ATMi. OVCAR4 were also very sensitive to SN38 and insensitive to ATMi but despite the lack of ATM, they exhibit no known mutations in these pathways^23^; however, these cells were still relatively resistant to ATRi.

It has been proposed that cells with ATM mutations will result in low expression of the ATM protein, and this has been used as a justification for scoring ATM by immunohistochemistry as a predictor of clinical response to ATRi^24,25^. Our data clearly indicate that this approach is not justified, as cell lines that have dysfunctional mutations have highly variable levels of ATM protein. Further, when we investigated potential ATM dysfunction in 5 of the lowest ATM expressors, we found that several appeared fully functional for ATM (Supplementary Table 1). The exceptions are OVCAR4 discussed above, and possibly HCT15, which have loss of CHK2. Hence, low levels of ATM protein do not correlate with ATM dysfunction.

### Detection of ATM by immunohistochemistry

**Figure 6:**
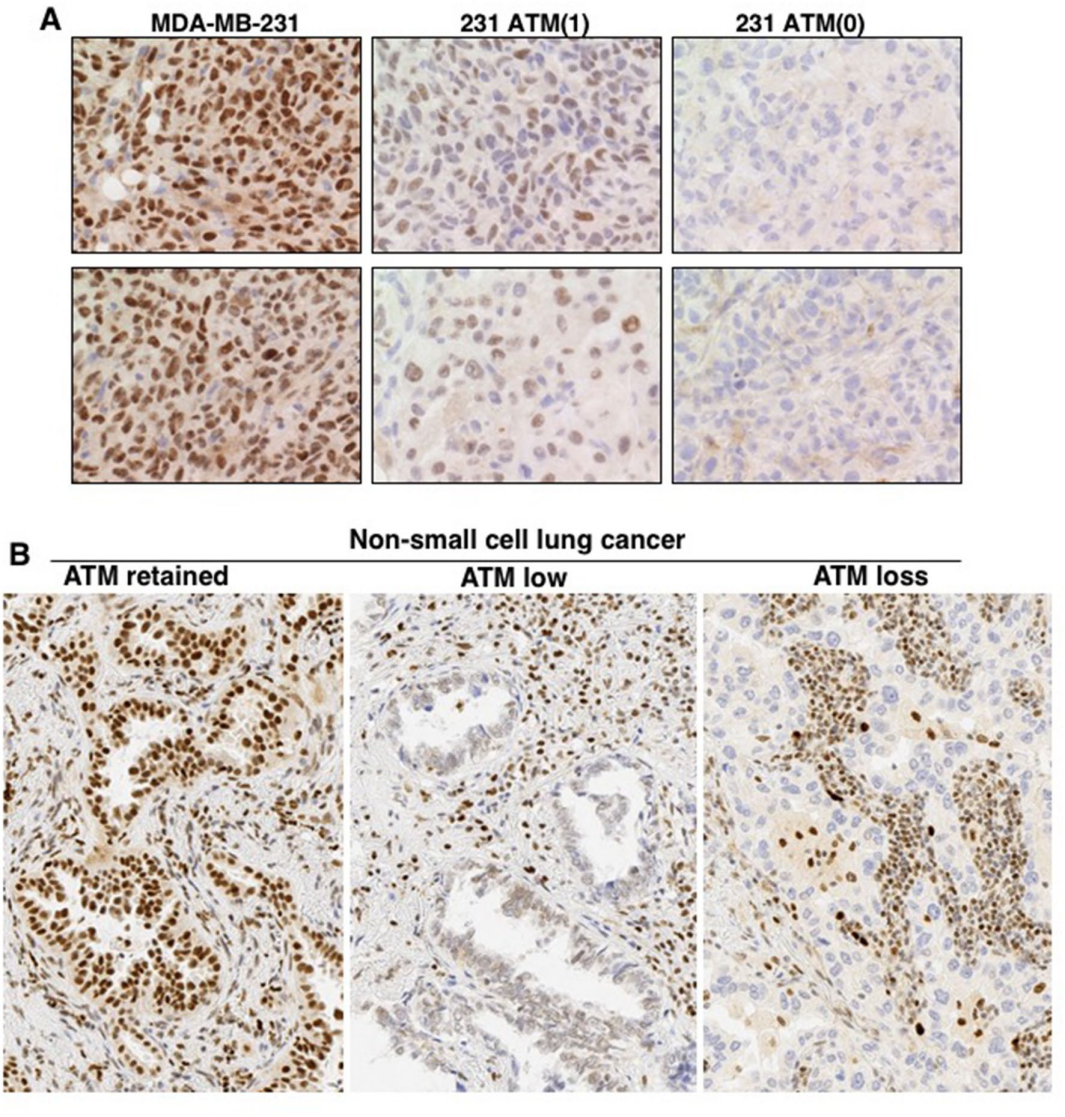
Immunohistochemistry of ATM in human tumor xenografts and non-small cell lung cancer. (**A**) MDA-MB-231 WT, 231 ATM(1), and 231 ATM(0) xenografts **w**ere generated and stained for ATM. Two fields are shown for each cell line (**B**) Non-small cell lung cancers were sectioned and stained for ATM. All fields were photographed at 20 × magnification. Representative pictures are shown for retained, low or loss expression. Each section also shows the staining of non-tumor tissue as a reference.

Our results highlight a concern for the use of immunohistochemistry (IHC) to assess ATM levels with the expectation that patients whose tumor has low ATM would respond to ATRi. In this regard, it has been variously reported that 9% or 41% of non-small cell lung cancers (NSCLC) are negative for ATM^24,25^. To address this discrepancy, we performed an IHC study using our MDA-MB-231 derivatives and a small panel of human NSCLC tumor samples. We first generated tumors in mice using our ATM deleted cells, and then scored them by immunohistochemistry (Fig. 6A). While the difference between the parent and ATM(0) was easy to distinguish, the level of ATM in the partial knockout was difficult to detect until we further optimized the IHC protocol, primarily involving a longer epitope retrieval time.

We also scored 21 human NSCLC tumors and found variable response (Fig. 6B). Five tumors appeared to have lost ATM, 10 had low ATM while 6 retained levels similar to the surrounding normal cells. Seven of these tumors had been selected for ATM mutations (all heterozygous), but only 2 of these had loss of ATM, while 3 scored as low. Whether any of the mutations were dysfunctional is unknown. We suspect that the report of 41% loss of ATM arose from an inadequately optimized protocol that failed to discriminate the low expressors, and thereby over-predicted the number of tumors that might be sensitive to ATRi.

### Sensitivity to ATRi depends on high CDK2 activity

The sensitivity of cells to CHK1i as a single agent can be prevented by addition of low concentrations of the CDK2 inhibitor CVT313. This was seen both as inhibition of γH2AX and recovery from growth inhibition and cell death^26,27^. At higher concentrations, CVT313 also prevents CHK1i-mediated S phase progression, γH2AX and replication catastrophe following incubation with DNA damaging agents^26,27^. This has led to the conclusion that different threshold activities of CDK2 are required for single agent activity versus its use in drug combinations^4^. These threshold activities likely depend on the particular cyclin partner, as suppression of cyclin A, but not cyclin E, protects cells from CHK1i^5^.

**Figure 7:**
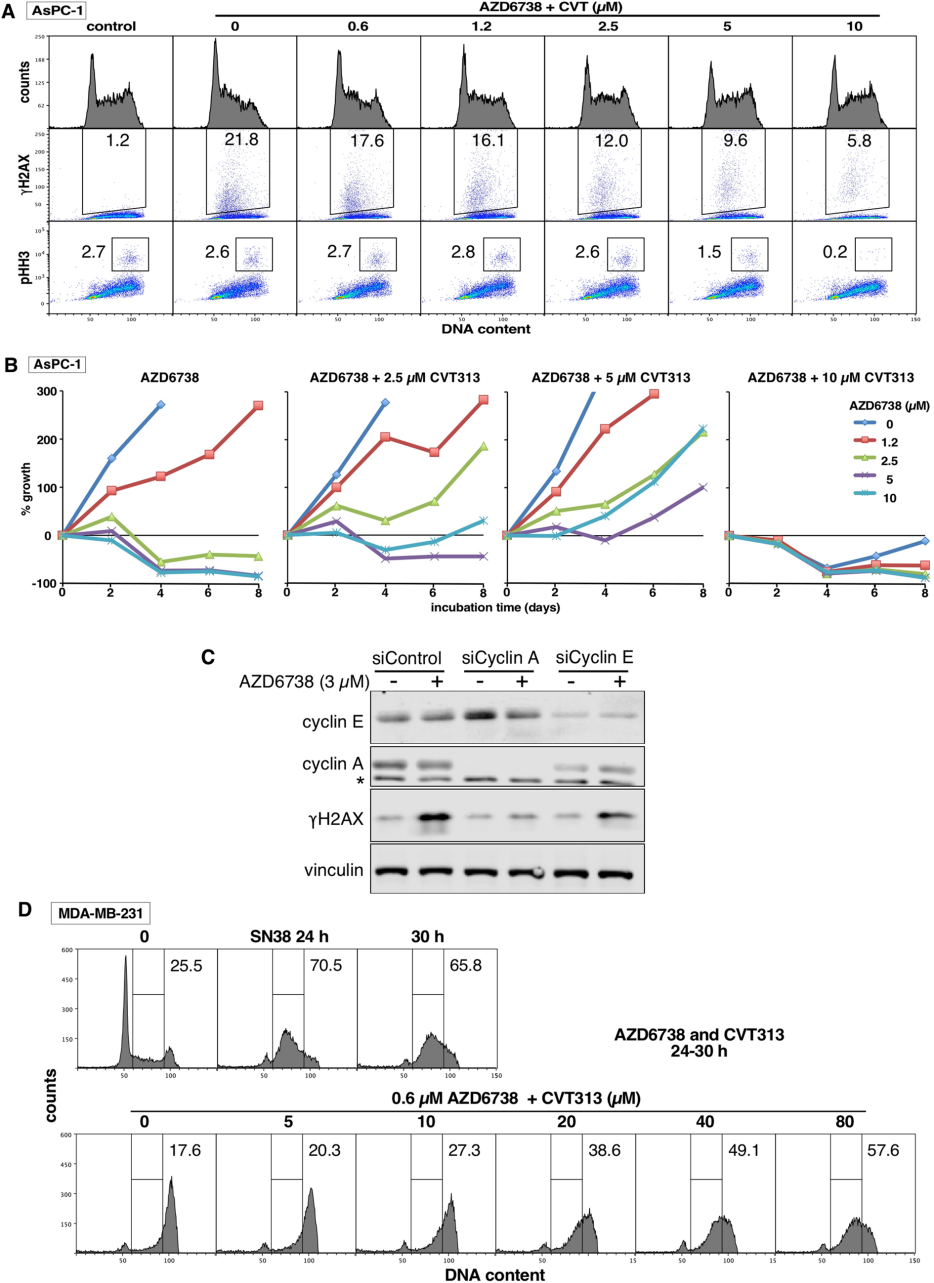
Dependence of γH2AX and sensitivity to ATRi on CDK2 and cyclin A. (**A**) AsPC-1 cells were incubated with 1 µM AZD6738 for 6 h, concurrent with 0–10 µM CVT313. Cells were harvested and analyzed by flow cytometry. This experiment was repeated in 4 additional cell lines as shown in Supplementary Fig. 5A. (**B**) AsPC-1 cells were plated at 10,000 cells/well, then with 0–10 µM AZD6738 concurrent with 0–10 µM CVT313 for 48 h. The drugs were removed and cells harvested every 2 days. Curves that descend below the starting inoculum reflect cell death. (**C**) AsPC-1 cells were transfected with siRNA targeting cyclin A, cyclin E or a control siRNA, then incubated with 3 µM AZD6738 for 24 h. Cell lysates were analyzed by western blotting. * = non-specific band. (**D**) MDA-MB-231 cells were incubated with 10 ng/mL SN38 for 24 h, the drug was removed, and further incubated with 0.6 µM AZD6738 concurrent with 0–80 µM CVT313. After an additional 6 h, cells were harvested, fixed and analyzed by flow cytometry. This experiment was repeated in PC3 cells as shown in Supplementary Fig. 5B.

Here, we confirm that low concentrations of CVT313 (1.2–2.5 µM) also prevent γH2AX induced by ATRi in AsPC-1 cells (Fig. 7A). Low concentrations of CVT313 also suppressed γH2AX in four other cell lines (Supplementary Fig. 5A). In addition, low concentrations of CVT313 also rescued AsPC-1 cells from cell death induced by ATRi (i.e., less than starting inoculum) and enhanced growth, while 10 µM CVT313 alone prevented growth (Fig. 7B). Protection from ATRi was also observed in a second sensitive cell line, U2OS (Supplementary Fig. 5B). CVT313 also inhibits CDK1 at higher concentrations, but the fact that the low concentrations did not arrest cell growth supports the conclusion that CDK1 is not inhibited at these concentrations. This is further confirmed by the observation that concentrations of CVT313 below 10 µM CVT313 did not inhibit mitosis as assessed by decreased pHH3 (Fig. 7A, Supplementary Fig. 5A). We also confirmed that suppression of cyclin A rather than cyclin E protected AsPC-1 cells from ATRi (Fig. 7C), as previously observed for CHK1i^5^.

In parallel, we compared the concentrations of CVT313 that prevented S phase progression when ATRi is added to damaged cells. This was performed in resistant cell lines as the DNA damage induced by single agent activity of ATRi exacerbates the S phase progression in sensitive cells. As discussed above (Fig. 3), ATRi still abrogates arrest in these cells, but high concentrations of CVT313 (20–80 µM) were required to circumvent the S phase progression (Fig. 7D, Supplementary Fig. 5B). Similar results have previously been obtained with CHK1i and, as discussed above, are attributed to a low threshold activity of CDK2 being required for S phase progression^26,27^. Replication stress also involves stalled replication forks that require a low threshold activity of CDK2 to restart replication (inhibited by high concentrations of CVT313) whereas the single agent activity of ATRi, like CHK1i, depends on high threshold activity of CDK2 (inhibited by low concentrations of CVT313). These observations further support our conclusion that endogenous replication stress does not explain the sensitivity of cells to either CHK1i or ATRi as single agents.

## Discussion

Variable sensitivity of cell lines to both CHK1i and ATRi has been frequently reported, and this has led to investigations into the underlying mechanisms with the goal of stratifying cancer patients most likely to respond to these drugs. Probably, the most frequently implicated reason for this sensitivity is the presence of endogenous replication stress^6–10^. Oncogene-induced stress is also frequently implicated in sensitivity to these drugs, yet this also involves endogenous replication stress^28,29^. However, while replication stress is a consequence of inhibition of CHK1 or ATR, our results do not support the contention that endogenous replication stress is a pre-requisite for sensitivity to checkpoint inhibitors. First, we established that there exists a very different profile of sensitivity to CHK1i and ATRi, hence replication stress cannot be the explanation for sensitivity to both drugs. Second, DNA damaging agents such as hydroxyurea or gemcitabine that induce replication stress are further sensitized by CHK1i or ATRi, and it has been assumed that this is the same mechanism for the single agent activity of CHK1i and ATRi. When used in combination with hydroxyurea or gemcitabine, sensitivity to CHK1i or ATRi results from reactivation of stalled replication forks, a process that requires only a low activity threshold of CDK2^26^. In this drug combination, the addition of CHK1i does not induce S phase progression because of the lack of dNTPs as a result of the inhibition of ribonucleotide reductase. However, when cells are arrested in S phase with the topoisomerase inhibitor SN38, the addition of CHK1i does induce S phase progression, and this also requires only a low level activity of CDK2^27^. We demonstrated a similar phenomenon here whereby ATRi-induced S phase progression after DNA damage by SN38 is inhibited only at high concentrations of CDK2i (i.e., low activity CDK2). In contrast, we previously established that the single agent activity of CHK1i is inhibited by low concentrations of CDK2i (i.e. high activity CDK2)^5,26^, and that is reiterated here for single agent activity of ATRi. In addition, we confirm that suppression of cyclin A protects cells from γH2AX induced by ATRi in sensitive cells, as previously reported for CHK1i^5^. Hence, the mechanism of single agent activity for both CHK1i and ATRi differs from the combination with hydroxyurea or SN38, and consequently does not require replication stress.

It has also been reported that sensitivity to both drugs results in premature mitosis^17,18^, yet if this were the case, there would be a strong correlation between the appearance of γH2AX and pHH3. In all the experiments reported here, we observed pHH3 only in the G2/M phase of the cell cycle, and it was not associated with the cells positive for γH2AX which were primarily in S phase. Premature mitosis can result when CDK1 is activated in S phase. That this did not occur concurrent with γH2AX is consistent with our observations that CDK2 is preferentially activated when cells are incubated with CHK1i^26,27^. Premature mitosis may be more relevant to incubation with a WEE1 inhibitor which may prematurely activate CDK1 in S phase cells^17,18^.

A previous study reported that the CHK1i UCN-01 induced far more γH2AX than ATRi^16^ leading to the assumption in subsequent papers that inhibition of CHK1 is more deleterious to cycling cells than inhibition of ATR^30,31^. This conclusion derived from an experiment in U2OS that compared only a single concentration of each drug, and a failure to realize that U2OS cells are one of few cell lines that are very sensitive to CHK1i as a single agent. Furthermore, UCN-01 has numerous off-target effects that might impact γH2AX. From our analysis of 65 cell lines, we confirm that U2OS cells are very sensitive to CHK1i (MK-8776), and only slightly less sensitive to ATRi (AZD6738) (Fig. 1). U2OS cells also require about fourfold higher concentration of AZD6738 than MK-8776 to achieve the same level of γH2AX (Fig. 2). A parallel experiment in AsPC-1 cells showed that a similar fourfold higher concentration of AZD6738 was required to induce γH2AX consistent with the difference in cytotoxicity.

However, we caution that γH2AX may not be a reliable predictor of sensitivity to ATRi as ATR and ATM both phosphorylate H2AX^32^, hence the use of an ATRi in the absence of ATM may result in limited γH2AX. We have observed this phenotype in both the 231 ATM(0) and H23 cells that lack functional ATM and are very sensitive to ATRi (Supplementary Fig. 6). Furthermore, ATRi inhibited the γH2AX induced by CHK1i in the H23 cells. Residual γH2AX may be due to activation of DNA-PK.

Another commonly proposed predictor of sensitivity to ATRi is mutation of ATM, which would put greater reliance on ATR for the DNA damage response. ATM is a very large gene that is frequently mutated in cancer but the mutations occur throughout the gene with no hotspots. This raises the complexity of determining which mutations might be dysfunctional, and also whether mutation of a single allele acts as a dominant negative. We first generated an isogenic cell line deleted for ATM, and found that it was indeed sensitive to ATRi. This cell line validated a functional assay for ATM dysfunction that relied on the sensitivity of cells to SN38: cells defective for ATM should be sensitive to SN38 and exhibit no further sensitization upon addition of an ATM inhibitor. Importantly, there was no correlation across multiple cell lines between ATM dysfunction in this assay and sensitivity to ATRi. Several cell lines had mutations in ATM that appeared dysfunctional, yet were still resistant to ATRi. Other cell lines appeared dysfunctional for ATM pathways but with no known mutations in ATM, and were also resistant to ATRi. This may be attributable to other defects in the ATM pathway. Alternately, many cell lines sensitive to ATRi exhibited no ATM dysfunction. At the current time, this approach may reflect the best available assay for ATM pathway dysfunction, but it is clear that it does not correlate with sensitivity to ATRi.

A concern extending from these observations is that investigators have proposed using immunohistochemistry of ATM to predict sensitivity to ATRi^24,25^. While generating the isogenic ATM knockout, we also generated an intermediate line with low level ATM but was still fully functional for ATM. This low level was difficult to detect by immunohistochemistry, and we suggest this may explain the discrepancy between the frequency of tumors reported to have lost ATM.

We also investigated whether other defects might occur in the DNA damage response. A few of the cell lines exhibited elevated basal phosphorylation of ATM, CHK2, ATR or CHK1. The addition of SN38 permitted assessment of whether the DNA damage response pathway was functional, and showed that a couple of cell lines had little to no activation. However, once again, there was no correlation between basal level activation of DDR dysfunction with sensitivity to ATRi or CHK1i.

Many other gene dysfunctions have been implicated in sensitivity to ATRi, using isogenic cell lines or genome-wide screens^33–36^. Many of these events were in DNA replication or repair and would have resulted in replication stress, which is inconsistent with our observations in sensitive cells. Furthermore, the purported sensitivity of ATM-defective cells has been tested in drug combinations, yet the impact of ATM could be on the DNA damaging agent (cisplatin) rather than ATRi^12^. In summary, some defects may elicit sensitivity in rare tumors, but no single defect appears to predict the sensitivity or resistance to ATRi or CHK1i in a significant number of tumors, so the current knowledge is of little value in stratifying patients for therapy. In contrast, we have demonstrated the importance of aberrant hyper-activation of CDK2 in eliciting sensitivity to both drugs and suggest that resolving the underlying mechanism may provide a better predictive prognostic marker.

## Methods

### Cell culture

The majority of cell lines were obtained from the Developmental Therapeutics Program of the National Cancer Institute as part of the NCI60 cell line panel. Other cell lines were obtained from the American Type Culture Collection. Cells were expanded and stored at low passage number and experimental cultures were replaced approximately every 3 months. Cells were maintained in RPMI1640 (Corning/Mediatech) plus 10% fetal bovine serum (Hyclone, Logan UT), and 1% antibiotic/antimycotic (Gibco, Carlsbad, CA). Cell lines were confirmed negative for mycoplasma using the MycoAlert Mycoplasma Detection Kit (Lonza).

### Chemicals

MK-8776 (Merck, Kenilworth, NJ), AZD6738 (Selleckchem, Houston, TX) and CVT313 (Sigma) were stored as a 10 mM solution in DMSO. No apparent change in potency of the stock solutions was observed over the course of this study.

### Growth inhibition assays

Cells were plated at 500–2000/cells per 100 µl in each well of a 96-well plate (depending on growth rate). The following day, drugs were added at twofold dilutions (8 wells per concentration). After 24 or 48 h, drug was removed, wells washed with phosphate buffered saline (PBS), and fresh media added. Alternately, drugs were left on the cells until harvest. Cells were harvested on day 6 or 7 when approaching confluence. Cells were lysed and DNA was stained with Hoechst 33258^21,37^. Fluorescence was read on a microplate spectrofluorometer. Results were expressed as the concentration that inhibited growth by 50% (GI50). The results for MK-8776 are derived from our prior publication with a few additions and deletions^5^. While it was impractical to repeat this screen in all cell lines, those selected for further analysis were reconfirmed in replicate experiments. As the starting cell number in this assay is very low, growth inhibition of 100% cannot discriminate between no growth (cytostasis) versus cell death. To assess cell death, cells were plated at 10,000 cells/well and harvested every 2 days for 8 days. This facilitated assessment of the decrease in cell number below the starting inoculum which equates to cell death^38^.

### Cell cycle analysis

Cell cycle analysis was conducted by flow cytometry using propidium iodide as described previously^39^. As required, cells were also labeled concurrently with Alexa 488-conjugated γH2AX and Alexa 647-conjugated pHH3 (Cell Signaling Technology, Danvers MA). Cells were analyzed on a Becton Dickinson Gallios flow cytometer. Data was analyzed using FlowLogic. All experiments were repeated in multiple cell lines to confirm that any particular phenotype was not unique to one cell line.

### siRNA

Cyclin A siRNA was Dharmacon On-Target Plus SMARTpool CCNA2 (L-003205-00-0005); cyclin E siRNA was Ambion Silencer Select CCNE1 (s2526); and the control siRNA was Ambion Silencer Select Negative Control #1 (4390843). Cells were transfected with 25 pmol siRNA using Invitrogen Lipofectamine RNAiMax transfection reagent according to manufacturer’s protocol. After 24 h, media was replaced and cells incubated in complete media with or without 3 µM AZD6738 for 24 h.

### Western blotting

Cells were rinsed in PBS, lysed in Laemmli lysis buffer, and boiled for 5 min. Protein levels in the lysates were determined by Pierce 660 nm Protein Assay Reagent with added Ionic Detergent Compatibility Reagent, and 40 µg was added to each lane, separated by SDS-PAGE and transferred to polyvinylidene difluoride membranes. Membranes were sectioned for appropriate regions such that multiple antigens of different sizes could be analyzed from a single blot. Western blotting was performed with the following primary antibodies: Cell Signaling Technology: pS1981-ATM (13050S), pT1989-ATR (58014S), pS345-CHK1 (2348S), pT68-CHK2 (2197S), γH2AX (9718S), PARP (9532S). Santa Cruz Biotechnology: CHK1 (sc8408), ATM (sc135663), ATR (sc15173). EMD Millipore: CHK2 (05-649). The following secondary antibodies were obtained from Cell Signaling technology mouse IgG-DyLight 800 (5257), rabbit IgG-DyLight 800 (5151), mouse IgG-DyLight 680 (5470). Fluorescent images of the desired region were captured using a Licor Odyssey imager and processed using Image Studio Lite. This approach provides a wide dynamic range that is directly quantified by the fluorescent scanner. The entire image captured is presented in each figure. PARP was used as a loading control while simultaneously confirming that apoptosis had not occurred. Given the large number of western blots in this screen, it was prohibitive to replicate all of them, but in cases where some unusual event was observed such as defective DNA damage signaling, no CHK2, no expression or over-expression of ATM, a new sample was analyzed to confirm the observation.

### Development of ATM-deficient cell lines

Optimal single guide RNAs (sgRNAs) targeting ATM exons 3 and 6 were selected using the online tool Benchling and obtained from Integrated DNA Technologies (Supplementary Fig. 4). Using the publicly available Zhang protocol (https://media.addgene.org/cms/filer_public/4f/ab/4fabc269-56e2-4ba5-92bd-09dc89c1e862/zhang_lenticrisprv2_and_lentiguide_oligo_cloning_protocol_1.pdf), ATM EX3 sgRNAs were inserted into the lentiCRISPRv2 puro vector (Addgene #98290), while ATM EX6 sgRNAs were inserted into the lentiCRISPRv2 blast vector (Addgene #98293).

For generation of lentivirus, 293-FT cells cultured in IMDM with 10% serum and geneticin were seeded at ~ 60% confluency in 10 cm plates. After 24 h, cells were co-transfected with 5 µg lentiCRISPRv2 puro ATMEX3 plasmid or lentiCRISPRv2 blast ATMEX6 plasmid, 3 µg psPAX2 packaging plasmid (Addgene #12260), and 2 µg pMD2.G envelope plasmid (Addgene #12259). The following day, medium was replaced with IMDM containing 2% serum and 2 mM caffeine. After 24 h, viral supernatant was harvested, filtered, and stored at − 80 °C.

Twenty-four h prior to transduction, MDA-MB-231 cells were seeded at 50% confluency in complete RPMI1640. Transduction was performed by replacing medium with 5 mL complete RPMI1640, 5 mL lentiCRISPRv2 puro ATMEX3 viral supernatant, and 1:1000 polybrene. Viral medium was replaced with complete RPMI1640 after 24 h. Cells were allowed to recover for 6 days, then selected for antibiotic resistance for 4 days in complete RPMI1640 supplemented with 2 µg/mL puromycin. Clonal cell lines were obtained using glass cloning rings. CRISPR efficacy was determined via Sanger sequencing of ATM exon 3 and western blotting for total ATM protein. A clone harboring one apparent functional copy of ATM exon 3 was maintained as the 231 ATM(1) cell line.

The 231 ATM(1) cells were further transduced with lentiCRISPRv2 blast ATMEX6 viral supernatant as described above and selected for 10 days in complete RPMI1640 with 10 µg/mL blasticidin. Clones were isolated as described above, and CRISPR efficacy was determined via Sanger sequencing of ATM exon 6 and western blotting for total protein. A clone expressing no detectable ATM protein was maintained as the 231 ATM(0) cell line.

### Immunohistochemistry

Animal studies were reviewed and approved by the Dartmouth College Animal Care and Use Committee (IACUC) and conducted in accordance with all applicable federal regulatory requirements, and in compliance with the recommended ARRIVE guidelines. Female NOD-scid/IL2Rg^-/-^(NSG; NOD.Cg-Prkdcscid Il2rgtm1Wjl/SzJ) mice were obtained from the Norris Cotton Cancer Center Mouse Modeling Shared Resource. Six mice (two for each cell line; 3–4 wk) were injected subcutaneously with 5 × 10^6^ MDA-MB-231, 231 ATM(1), or 231 ATM(0) cells. Tumor dimensions were measured twice weekly using calipers (volume = [length^2^ × width]/2). When tumors reached ~ 400 mm^3^, they were harvested, fixed in formalin, and embedded in paraffin.

Human non-small cell lung cancer specimens were obtained from archived tumor samples in the Research Pathology biospecimen bank, and in accordance with a protocol approved by the Dartmouth College Institutional Review Board. The tumor DNA had been analyzed by partial exon sequencing as part of standard molecular genotyping, albeit only 16 of 66 exons of ATM had been sequenced.

Tumor sections were cut at 4 µm, mounted on slides, and air dried at room temperature for at least 30 min. The slides were loaded on to a Leica Bond automated immunostainer, baked, dewaxed and incubated with BOND epitope retrieval solution 2 (pH 9; Cat. # AR9640) for 20 min at 100 °C. Primary antibody binding used the Bond Polymer Refine Detection Kit (DS9800) and anti-ATM antibody (Abcam: ab32420), and incubated for 30 min at 1:150 dilution (0.8 µg/mL). Primary antibody binding was visualized using Leica Bond Refine Detection kit with DAB chromogen and hematoxylin counterstain.

### Statistical analysis and replicates

The large cell line screens presented here were performed to identify differences in drug sensitivity or protein expression. For the candidate cell lines selected for further study, their sensitivity or resistance was confirmed. Similarly, outlier responses in the DNA damage response pathway were also confirmed by replicate western blots. The results of the protein analyses were further compared statistically for correlations. GraphPad Prism 8 was used to generate box-and-whisker plots of SN38-induced fold change of each antigen in ASPC-1 cells (n = 18). Whiskers represent minimum and maximum values, boxes represent 25th–75th percentiles, and lines represent the median for each antigen (Fig. 4B). For Supplemental Fig. 3, GraphPad Prism 8 was used to calculate non-parametric Spearman correlation coefficients for each plot (two-tailed P values, 95% confidence interval). For other experiments, replicates were performed in multiple cell lines to demonstrate reproducibility of a particular observation.

## Supplementary Information

Supplementary Information 1.

Supplementary Information 2.

Supplementary Information 3.
