# Supplementary material for: Sensitivity of cells to ATR and CHK1 inhibitors requires hyperactivation of CDK2 rather than endogenous replication stress or ATM dysfunction
Source: Sci Rep. 2021 Mar 29;11:7077. doi: 10.1038/s41598-021-86490-x (PMC8007816; doi:10.1038/s41598-021-86490-x)
Supplement: Supplementary file 1 — Supplementary Information 1. [file 41598_2021_86490_MOESM1_ESM.pdf]

Sensitivity of cells to ATR and CHK1 inhibitors requires hyper-activation of CDK2 rather than endogenous replication stress or ATM dysfunction

Jennifer P. Ditano, Katelyn L. Donahue, Laura J. Tafe, Charlotte F. McCleery, Alan Eastman

Department of Molecular and Systems Biology, and Norris Cotton Cancer Center, Geisel School of Medicine at Dartmouth, Lebanon NH 03756

Supplementary Table and Figures 1-3

Supplementary Table 1 (next page): Summary of data sorted by ATM levels (Fig. 4), compared to toxicity data (Fig. 1), known ATM mutations with SIFT and polyphen scores, other related defects, sensitivity to SN38 and sensitization to SN38 by ATMi. MDA-MB-231 and is derivatives are highlighted in red. Toxicity data is shown red for sensitive (GI50 < 1) or green for resistant (GI50 >7.5). This data is also provided as an Excel spreadsheet in the supplementary information.

Sensitivity of cells to ATR and CHK1 inhibitors requires hyperactivation of CDK2 rather than endogenous replication stress or ATM dysfunction

Jennifer P. Ditano, Katelyn L. Donahue, Laura J. Tafe, Charlotte F. McCleery, Alan Eastman

Table S1

cell line	ATM protein	ATRi GI50 (24 h)	ATRi G!50 (48 h)	TRi GI50 (continuous	CHKi GI50 (24 h)	CHKi G!50 (48 h)	CHKi GI50 (continuous)	known aberrations	ATM mutations	SIFT	Polyphen2	GI50 SN38	GI50 SN38+ATMi	fold
231-ATM(0)	0	0.5	0.3	0.3	10	0.4	0.4		ATM knockout			0.2, 0.4	0.15, 0.3	1.3
OVCAR4	0.0	10	2.0	1.5	10	1.0	0.6		no detetable ATM			0.12	0.1	1.2
SKBR3	0.1	3.7	0.9	0.6	6.0	0.6	0.3							
ACHN	0.1	1.3	0.6	0.3	0.2	0.2	0.09	(low ATR)				1.2	0.2	6
HT29	0.1	3.8	2.4	2.4	10	10	7.5					0.4	0.12	3.3
HCT15	0.1	2.5	1.3	1.0	10	10	0.3	CHK2 loss				3.5	1.5	2.3
CAPAN1	0.2	1.8	1.3	0.5	10	2.0	1.0	**BRCA2 defect**				2, 2.5	0.12, 0.3	16.7
MCF7	0.2	5.0	1.3	0.9	10	10	7.5					0.3	0.25	1.2
HCC1937	0.2	2.5	1.3	1.3	10	10	10							
BT549	0.2	3.8	0.9	0.9	10	10	7.5							
231-ATM(1)	0.2	2	1.0	1.0	10	1.5	1.5							
A498	0.2	0.3	0.5	0.5	0.2	0.2	0.1							
A2780	0.2	0.9	0.5	0.5	0.6	0.3	0.1							
RXF393	0.3	0.9	0.9	0.9	0.6	0.2	0.2							
HCT116	0.3	10	7.0	5.0	10	10	2.0	Mre11 protein low	D1853N (41%)	0.04	0.251	0.3	0.1	3
COLO205	0.3	0.6	0.2	0.3	10	10	7.5							
H1299	0.3	3	2.0	2.0	0.5	0.4	0.4							
T47D	0.3	10	10	5.0	10	10	3.0		D1853N (46%)	0.04	0.251	0.8	0.2	4
NCI H23	0.4	0.6	0.2	0.2	10	0.6	0.4		Q1919P (homo)	?	1	0.3	0.12	2.5
U87	0.4	0.7	0.3	0.2	0.5	0.2	0.2							
SKOV3	0.4	2.5	10.0	6.0	10	2.5	0.75		D1853N (45%)	0.04	0.251			
NCI H322M	0.5	0.9	0.2	0.2	10	10	2.0					0.5	0.08	6.25
IMEC	0.5	10	5.0	3.8	10	10	6.0					1.2	0.5	2.4
UACC62	0.5	10	10	2.5	10	6	0.6		P1054R (70%)	0	0.975			
786-0	0.5	1.9	0.9	0.9	10	3.8	0.6		R2436S (40%)	0.02	0.805	0.5	0.12	4.2
TK10	0.5	1.3	0.7	0.4	1.0	0.6	0.3	lowest ATM mRNA	**P1054R (100%)**	**0**	**0.975**	3	2	1.5
2008	0.5	0.3	0.3	0.2	10	10	10	FANCD2-Ub defect						
DU145	0.6	2.5	1.9	1.9	10	10	1.5							
SF539	0.6	1.9	1	0.9	5	0.9	0.6							
OVCAR5	0.6	2.5	1.9	1.9	10	2	0.5		D1853N (23%)	0.04	0.251	0.5	0.2	2.5
MDA-MB-231	0.7	1.9	0.6	0.6	10	2.5	1.8					1.25, 1.5	0.15, 0.2	8.4
LOXIMVI	0.8	0.9	0.6	0.6	10	10	3							
A549	0.8	10	5	3	10	10	3							
2008 FANCF	0.8	3	1.3	1	10	10	4							
OVCAR8	0.9	10	3.8	2.8	10	10	3		V613L (36%); D1853N (47%)	0.18/0.04	0.98/ 0.251	0.15	0.07	2.1
CAKI1	0.9	1.3	0.5	0.4	10	3.8	0.6		L2307F (100%)	0.04	0.308	0.9	0.15	6
AsPC-1	1.0	1.5	0.9	0.4	0.5	0.2	0.2					3.5	1.5	2.3
IGROV	1.0	10	3.8	1.3	10	1.3	0.9		R248Q (38%)	0	1	0.9	0.2	4.5
ATLD1-MRE11	1.0	2.5	1.3	1.9	10	10	3							
HOP62	1.1	1.9	0.9	0.9	10	10	1							
SNB19	1.1	10	7.5	7.5	10	10	10							
SN12C	1.2	10	10	10	10	10	7.5							
H460	1.2	1.9	1.1	0.7	10	8	5							
MDA-MB-468	1.3	2.5	0.9	0.6	10	4	3					0.25	0.08	3.1
HCC2998	1.3	0.6	0.3	0.3	3	1.2	1.2		R337C(53%)	0	1			
ADR-RES	1.3	10	10	10	10	10	10							
MiaPaCa-2	1.5	5	0.9	0.5	10	10	10							
NCI H522	1.6	7.5	2.5	1.9	10	10	3		**P1054R (27%)**	**0**	**0.975**	0.2	0.1	2
SW620	1.7	0.5	0.2	0.2	10	10	10					0.7	0.1	7
ATLD1	1.8	10	10	10	10	10	10	Mre11 defect						
M14	2.2	2.5	1.3	1.3	10	4	2.3							
PC3	2.3	10	10	2.5	10	10	7.5					1	0.2	5
U2OS	2.9	2.5	2.5	2	1.2	0.5	0.5					4	1	4
MDA-MB-435	2.9	2.0	1.0	0.8	1.0	0.2	0.2							
U251	3.5	2.5	1.9	1.9	10	10	7.5	Mre11 protein low	D1853N (41%)	0.04	0.251	0.25	0.1	2.5
UACC257	4.0	10	5.0	2.5	10	10	10							
EKVX	4.7	10	5	5	10	10	10	5.7x ATM gene						
UO31	5.3	10	1.9	1.9	10	7.5	0.8							
SF268	7.4	10	5	2.5	10	10	10	8.7x ATM gene	D1853N (90%)	0.04	0.251	0.12	0.1	1.2
	values as in Fig. 4							Mre11 from ref 19	discover.nci.nih.gov/cellminer	SIFT cut off is 0.05		sensitive		minimal ATMi sensitization

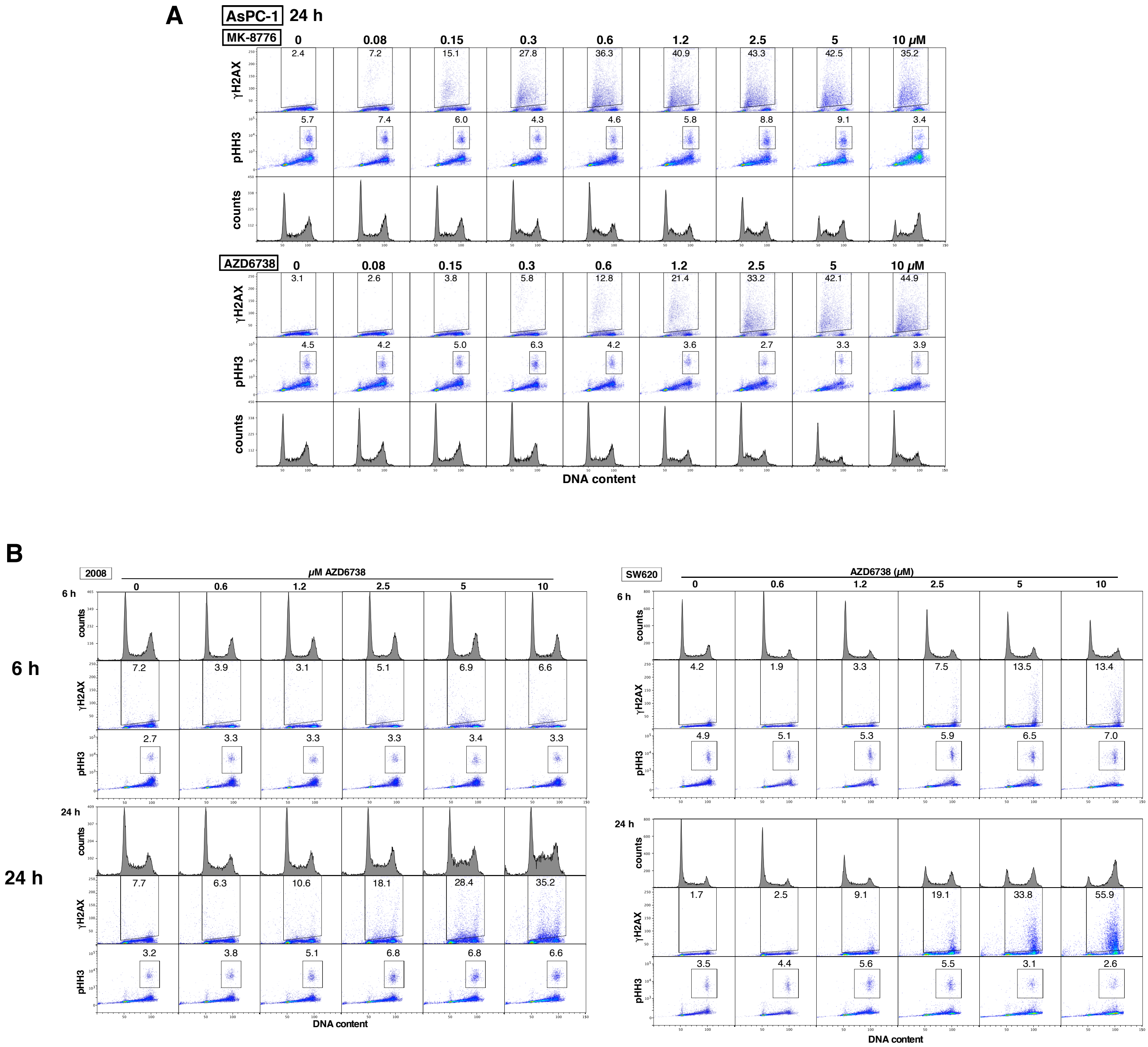

Supplementary Figure 1: Induction of γH2AX by AZD6738 and MK-8776 in additional cell lines. This Figure represents data summarized in Fig. 2. **A.** Comparison of MK-8776 and AZD6738 n AsPC-1 cells after 24 h incubation. **B.** Comparison of 2008 and SW620 cells incubated with the indicated concentrations of AZD6738 for either 6 h or 24 h, then fixed and analyzed by flow cytometry for DNA content, γH2AX and pHH3. Inset numbers represent the percentage of cells positive for γH2AX or pHH3.

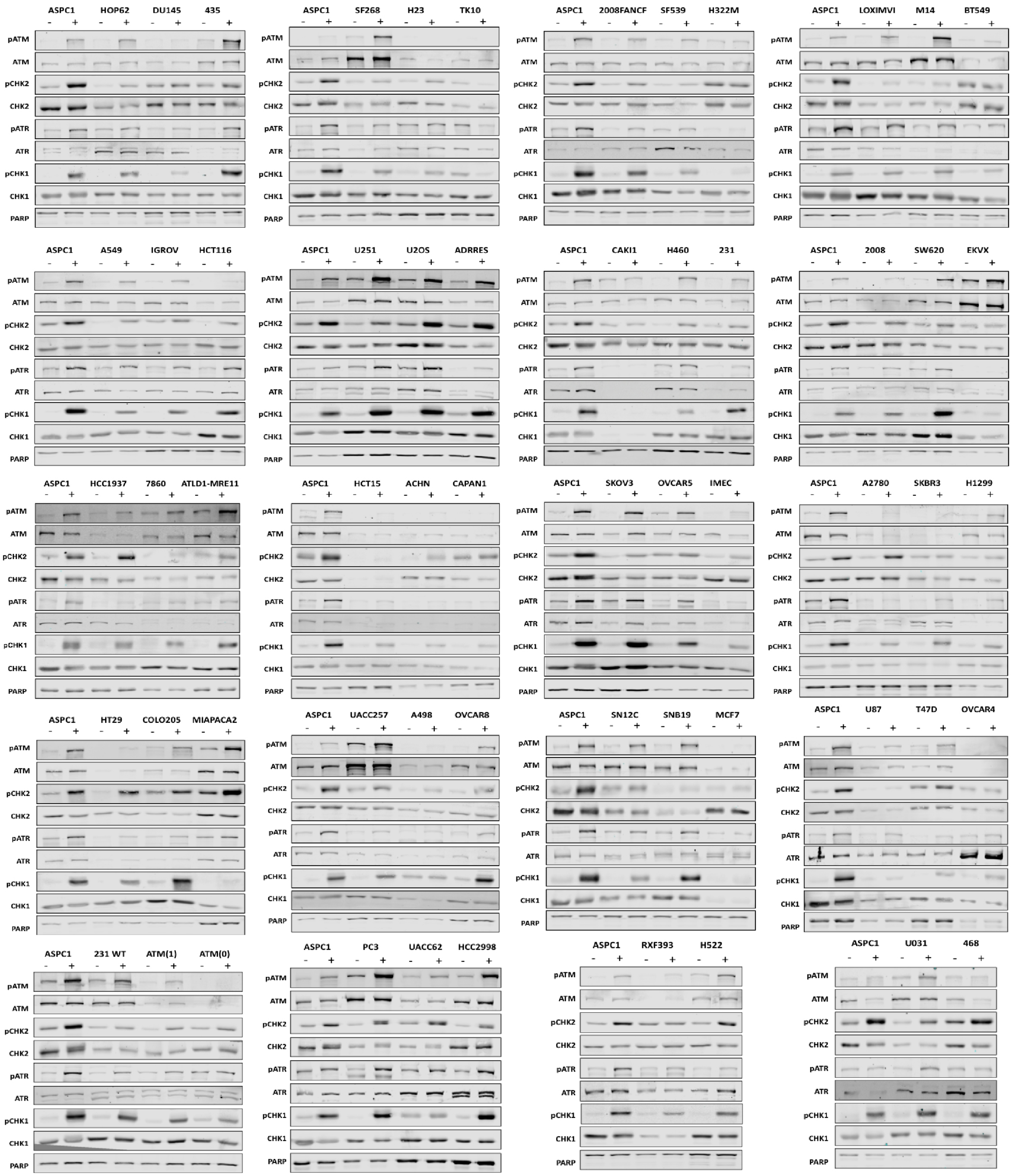

Supplementary Figure 2: Basal and SN38-induced DDR pathway markers across multiple cell lines. The indicated cell lines were untreated or incubated with 10 ng/mL SN38 for 6 h, then lysed and analyzed using the indicated primary antibodies and fluorescent secondary antibodies. Fluorescent images were generated using a Licor Odyssey imager and processed using Image Studio Lite. Each individual panel includes the internal standard cell line (AsPC-1) and 3 additional cell lines. Quantitation of the fluorescent signals was used to generate the data presented in Fig. 4.

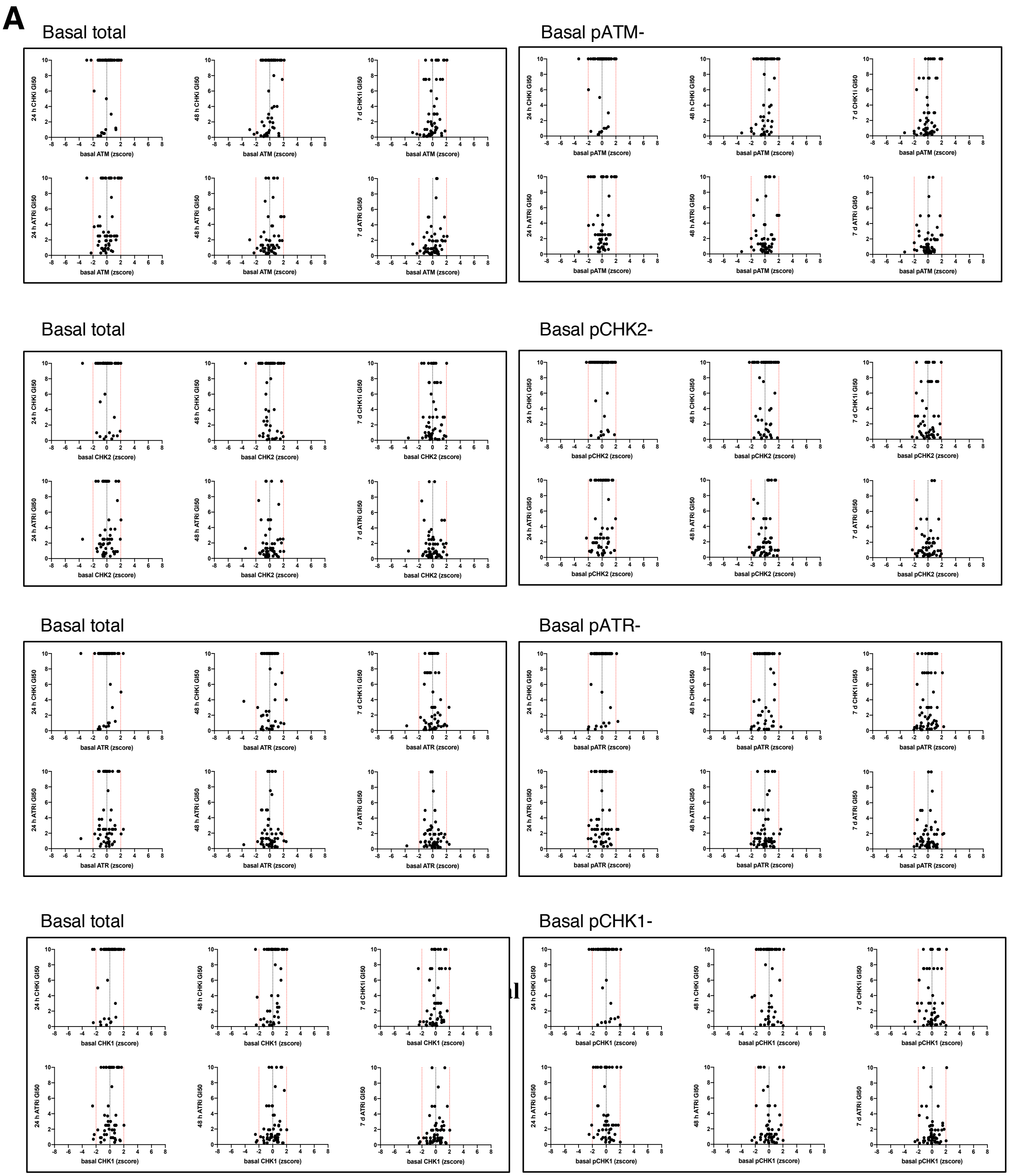

Supplementary Figure 3A (legend next page)

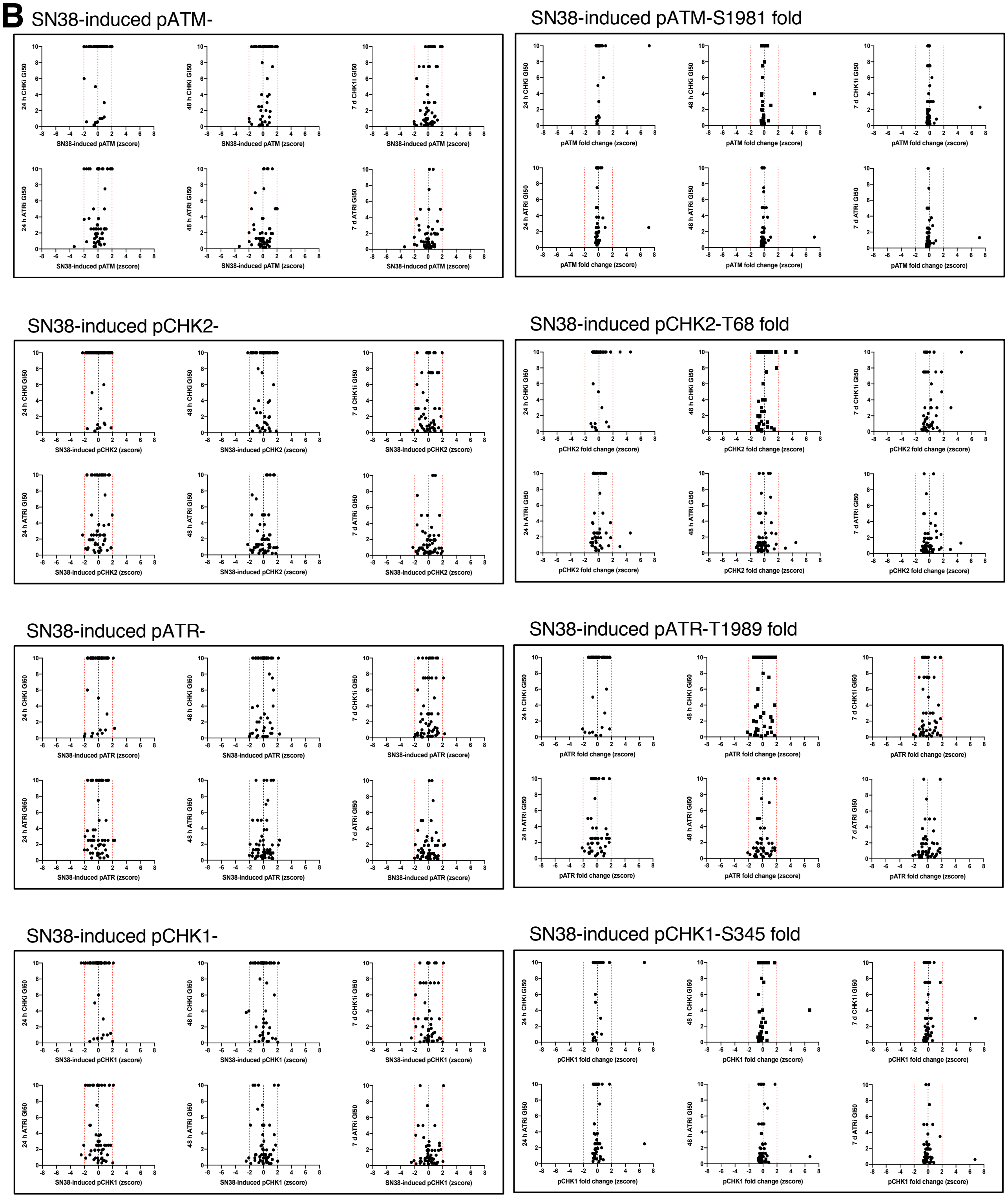

Supplementary Figure 3: Correlation between sensitivity to MK-8776 or AZD6738 and basal or SN38-induced protein levels. A. For the indicated protein or phospho-protein, basal protein z-score of each cell line was plotted against the GI50 for MK-8776 (CHK1i) or AZD6738 (ATRi) at 24 h, 48 h or 7 days in the same order as plotted in Fig. 1A. **B.** For the indicated phospho-protein, the z-score of SN38-induced protein level (left) or fold change (right) of each cell line was plotted against the GI50 for MK-8776 (CHK1i) or AZD6738 (ATRi) at 24 h, 48 h or 7 days in the same order as plotted in Fig. 1B.
