# Supplementary material for: Sensitivity of cells to ATR and CHK1 inhibitors requires hyperactivation of CDK2 rather than endogenous replication stress or ATM dysfunction
Source: Sci Rep. 2021 Mar 29;11:7077. doi: 10.1038/s41598-021-86490-x (PMC8007816; doi:10.1038/s41598-021-86490-x)
Supplement: Supplementary file 2 — Supplementary Information 2. [file 41598_2021_86490_MOESM2_ESM.pdf]

Sensitivity of cells to ATR and CHK1 inhibitors requires hyper-activation of CDK2 rather than endogenous replication stress or ATM dysfunction

Jennifer P. Ditano, Katelyn L. Donahue, Laura J. Tafe, Charlotte F. McCleery, Alan Eastman

Department of Molecular and Systems Biology, and Norris Cotton Cancer Center, Geisel School of Medicine at Dartmouth, Lebanon NH 03756

Supplementary Figures 4 - 6

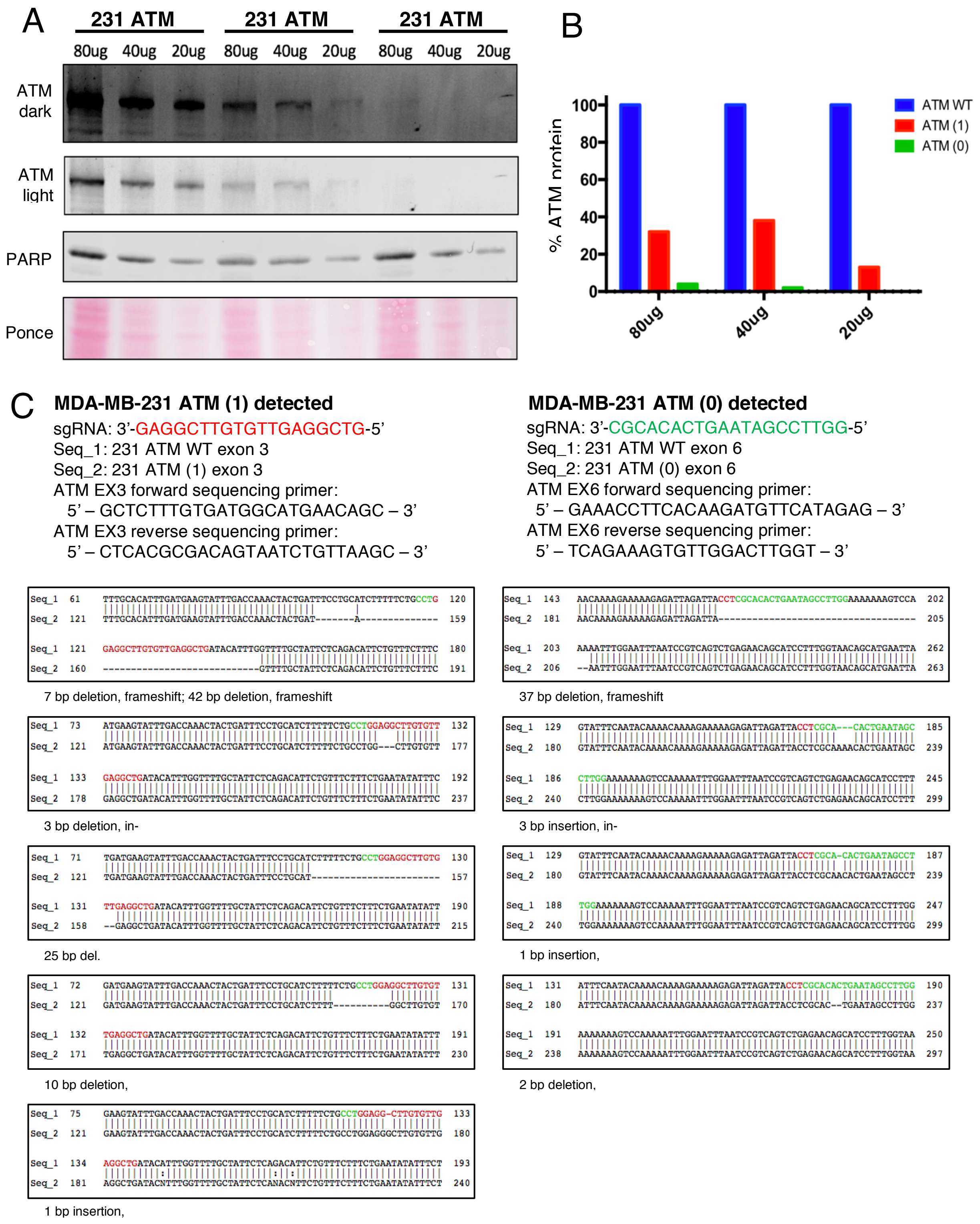

Supplementary Figure 4: Generation of MDA-MB-231 cells with partial or complete ATM loss. A. Wild-type [231 ATM WT], partial knockout [231 ATM(1)] and complete knockout [231 ATM(0)] cells were rinsed in PBS, lysed in Laemmli lysis buffer, and 20 – 80 µg protein analyzed by western blotting using ATM primary antibody and fluorescent secondary antibodies. Images were generated using a Licor Odyssey imager and processed using Image Studio Lite. Ponceau stain of the same region of the western blot was obtained prior to incubation with primary antibody. **B.** Signal intensity values from A were plotted for each derivative at the indicated protein concentrations. **C.** The DNA sequences from each unique allele for MDA-MB-231 ATM(1) and ATM(0) [derived from ATM(1)]. "Frame shift" indicates a likely deleterious mutation while "in-frame" indicates a potentially tolerated mutation.

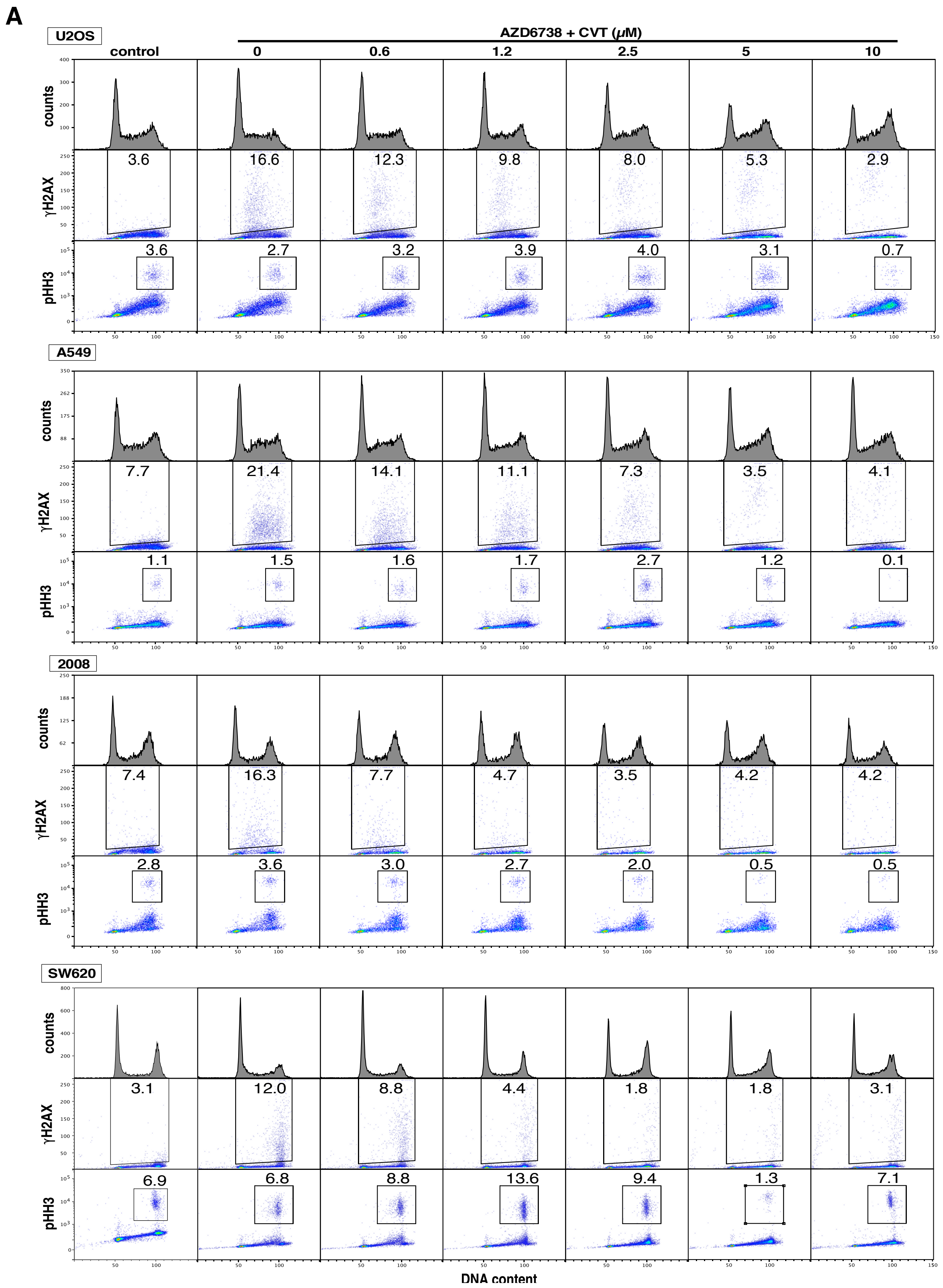

Supplementary Figure 5A (legend next page)

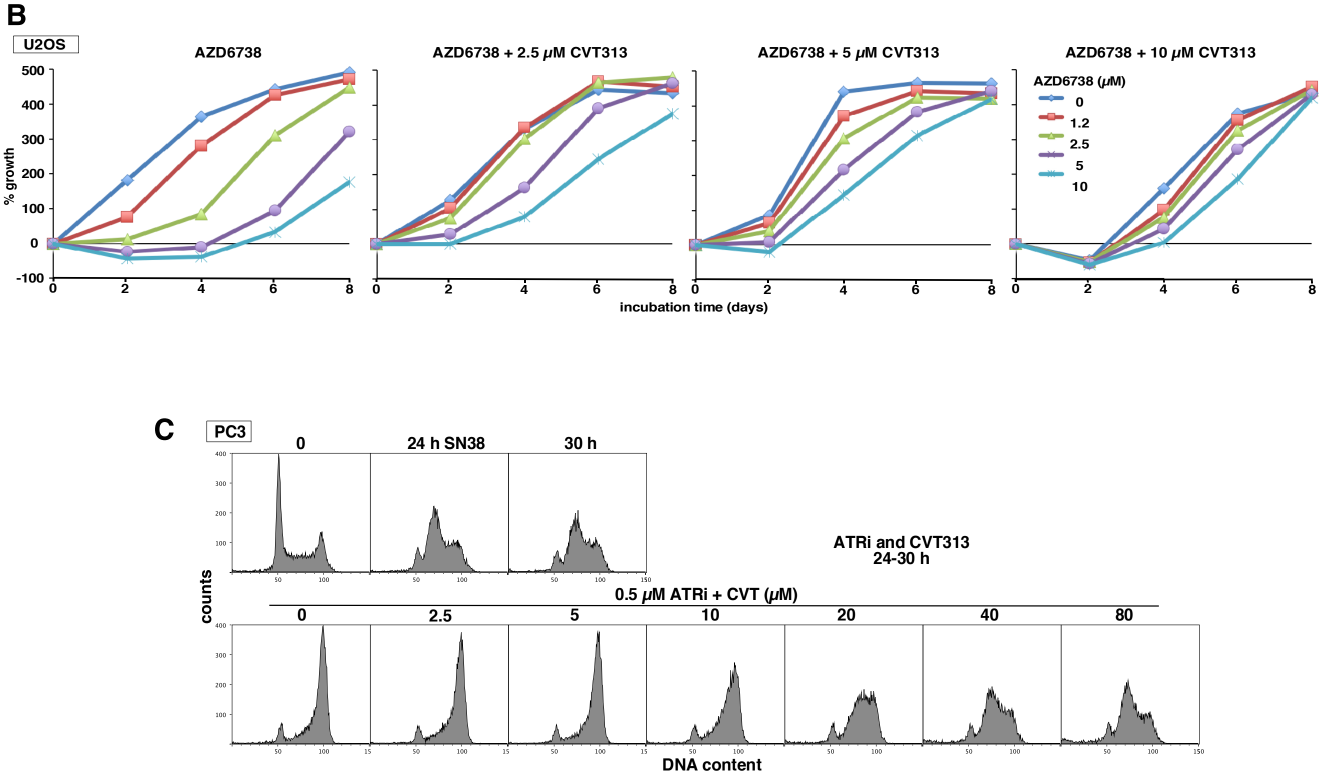

Supplementary Figure 5: Dependence of γH2AX and sensitivity to ATRi on CDK2. These experiments are a repeat of experiments in Fig. 7 except in additional cell lines. **A.** Four cell lines were incubated with 1 μM (U2OS) or 10 μM AZD6738 for 6 h, concurrent with 0 – 10 μM CVT313. Cells were harvested and analyzed by flow cytometry. **B.** U2OS cells were plated at 10,000 cells/well, then with 0 – 10 μM AZD6738 concurrent with 0 – 10 μM CVT313 for 24 h. The drugs were removed and cells harvested every 2 days. Curves that descend below the starting inoculum reflect cell death. Note that the parallel experiment in AsPC-1 cells in Figure 7B involved a 48 h concurrent incubation which may be why the U2OS cells here show less cell killing, and greater recovery after 10 μM CVT-313. **C.** PC3 cells were incubated with 10 ng/ml SN38 for 24 h, the drug was removed, and further incubated with 0.5 μM AZD6738 concurrent with 0 – 80 μM CVT313. After an additional 6 h, cells were harvested, fixed and analyzed by flow cytometry.

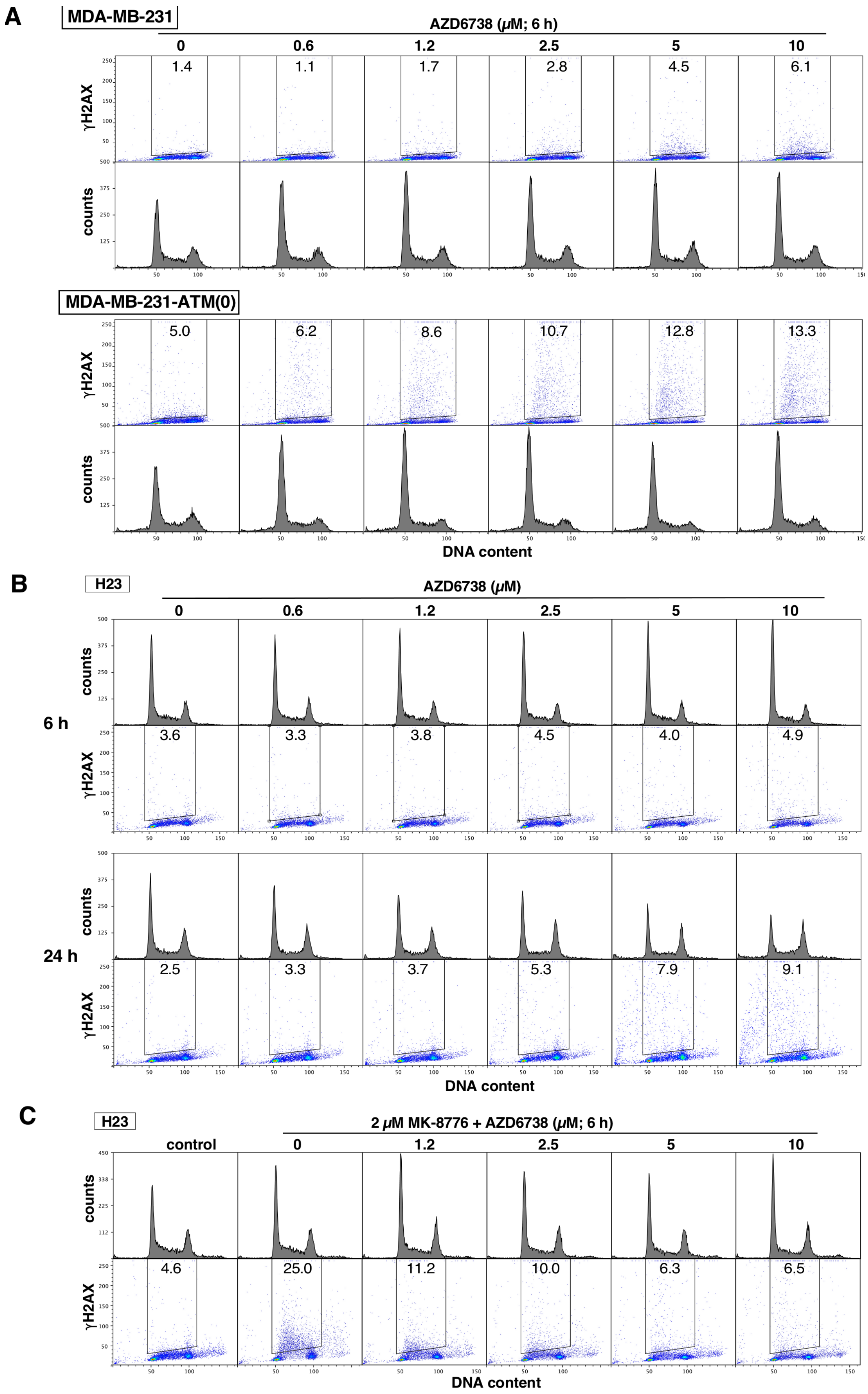

Supplementary Figure 6: Limitation to the detection of γH2AX in cells defective for ATM. A. MDA-MB-231 and 231ATM(0) cells were incubated with 0 – 10 μM AZD6738 for 6 h then fixed and analyzed by flow cytometry. **B.** H23 cells were incubated with 0 – 10 μM AZD6738 for 6 and 24 h then fixed and analyzed by flow cytometry. **C.** H23 cells were incubated with 2 μM MK-8776 for 6 h concurrent with 0 – 10 μM AZD6738 then fixed and analyzed by flow cytometry.
